# Amino Acid‐Sensing Neurons in the Anterior Piriform Cortex Control Brown Adipose Tissue Thermogenesis

**DOI:** 10.1002/advs.202502421

**Published:** 2025-04-30

**Authors:** Peixiang Luo, Kexin Tong, Yeting Gan, Min Tang, Yuguo Niu, Kan Liu, Shihong Ni, Shangming Wu, Xiaoxue Jiang, Haizhou Jiang, Fei Xiao, Shanghai Chen, Wei Lv, Xiaoying Li, Feixiang Yuan, Feifan Guo

**Affiliations:** ^1^ Department of Endocrinology and Metabolism Zhongshan Hospital Fudan University Shanghai China; ^2^ Institute for Translational Brain Research State Key Laboratory of Medical Neurobiology MOE Frontiers Center for Brain Science Fudan University Shanghai China; ^3^ Shanghai Institute of Nutrition and Health University of Chinese Academy of Sciences Chinese Academy of Sciences Shanghai China

**Keywords:** amino acid‐sensing, anterior piriform cortex, body temperature regulation, brown adipose tissue thermogenesis, corticotropin‐releasing hormone neurons

## Abstract

Amino acid sensing in the central nervous system plays a key role in regulating energy homeostasis. The anterior piriform cortex (APC) has been implicated in sensing amino acid deficiency and rapidly inducing an aversive response. However, the precise types of neurons involved and whether they possess additional metabolic regulatory functions remain to be elucidated. The study reveals that corticotropin‐releasing hormone (CRH) neurons in the APC (APC^CRH^ neurons) are activated by a leucine‐deficient diet to modulate brown adipose tissue thermogenesis and that they regulate body temperature in response to leucine deprivation. The findings reveal that APC^CRH^ neurons are sensitive to leucine‐deprivation signaling, with general control nonderepressive‐2 playing an essential role in enhancing their intrinsic excitability. Furthermore, APC^CRH^ neurons project into the known hypothalamic thermoregulatory region of the lateral hypothalamus, and APC^CRH^–lateral hypothalamus circuits mediate leucine deprivation‐induced thermogenesis. Additionally, it is observed that thermogenic regulation by APC^CRH^ neurons contributes to the maintenance of body temperature under cold exposure. Collectively, the findings identify a population of leucine‐sensing APC^CRH^ neurons, and reveal the signals and circuits involved in their regulation of brown adipose tissue thermogenesis and their subsequent contribution to body temperature regulation and energy homeostasis.

## Graphical abstract

This study identifies a class of amino acid‐sensing neurons, APC^CRH^ neurons, which regulate BAT thermogenesis. Leucine deficiency alters intrinsic excitability through GCN2 signaling pathway, activating APC^CRH^ neurons, which in turn regulate BAT thermogenesis via projections to the lateral hypothalamus. This finding provides new insights into the central regulation of peripheral metabolism by nutrient sensing.

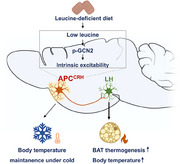

## 1 Introduction

The capacity of the central nervous system to gather information regarding the body's nutritional state and enact suitable behavioral and metabolic responses to fluctuations in energy availability is essential for maintaining metabolic homeostasis.^[^ ^1^, ^2^, ^3^ ^]^ The amino acids not only serve as essential building blocks for the body, but also convey a variety of signals through their fluctuating levels.^[^ ^4^, ^5^ ^]^ Previous studies have reported that the brain regions responsible for sensing amino acids are primarily located in the brainstem and hypothalamus.^[^ ^6^, ^7^, ^8^, ^9^ ^]^ These brain regions integrate nutritional signals to modulate food intake and/or peripheral metabolic processes,^[^ ^10^, ^11^, ^12^ ^]^ including the regulation of thermogenesis in adipose tissue.^[^ ^13^ ^]^

Given that the brain comprises intricate neural networks, elucidating the functions of specific amino acid‐sensing neurons is of substantial significance. With the elucidation of neurochemical properties over the past few decades, substantial progress has been made in identifying nutrient‐sensing neurons.^[^ ^14^, ^15^, ^16^ ^]^ Further studies have identified more specific neuronal types that sense amino acids, including agouti‐related peptide/neuropeptide Y and pro‐opiomelanocortin neurons in the arcuate nucleus and orexin/hypocretin neurons in the lateral hypothalamus (LH).^[^ ^17^, ^18^ ^]^ However, to date, little work has been done to target and identify amino acid‐sensing neurons outside these brain regions.

In addition to the brainstem and hypothalamus, the anterior piriform cortex (APC) has been identified as a key amino acid sensor within the brain.^[^ ^19^, ^20^ ^]^ It is shown that deficiencies in essential amino acids can independently activate the APC and inhibit the consumption of food lacking essential amino acids while promoting the search for more nutritionally balanced food options.^[^ ^20^, ^21^, ^22^ ^]^ However, the specific neurons implicated in this process have not yet been demonstrated. In addition to regulating food preferences, it is likely that the APC contributes to other phenotypes associated with essential amino acid deprivation. Therefore, it is necessary to identify the specific neuron types that sense amino acids and explore their functional involvement in metabolic control.

The amino acid responsive pathway is activated when essential amino acids are deficient. The general control nonderepressive‐2 (GCN2) kinase acts as a sensor of amino acid deprivation.^[^ ^23^, ^24^ ^]^ GCN2 detects intracellular amino acid levels by binding to uncharged tRNA. In the event of amino acid deficiency, the cellular levels of uncharged tRNA increase, triggering GCN2 kinase activation and subsequent phosphorylation at the serine 51 site of eukaryotic translation initiation factor 2 subunit 1 (eIF2α). Phosphorylation of eIF2α reduces protein synthesis and regulates gene expression at the transcriptional level.^[^ ^25^ ^]^ Furthermore, GCN2 is highly expressed in the brain and has been found to regulate circadian rhythms.^[^ ^26^, ^27^ ^]^ Considering its unreported involvement in central thermoregulation, it is plausible that the GCN2 present in the APC may be implicated in brown adipose tissue (BAT) thermogenesis.

In this study, we identify the corticotropin‐releasing hormone (CRH) neurons in the APC (APC^CRH^ neurons) as leucine‐sensing neurons, and elucidate their roles in detecting leucine deficiency through GCN2, and modulate BAT thermogenesis. Moreover, we reveal that these neurons are crucial for maintaining body temperature during cold exposure.

## 2 Results

### 2.1 APC Glutamatergic Neurons Modulate BAT Thermogenesis in Response to Leucine Deprivation

Leucine deficiency induces multiple beneficial metabolic effects, including increased BAT thermogenesis.^[^ ^28^, ^29^, ^30^ ^]^ Although the APC is a key component in sensing amino acid deficiency and inhibiting food intake, further research is needed to explore its other regulatory functions. To examine whether the APC plays a role in the BAT thermogenesis induced by leucine deprivation, we administered a leucine‐deficient diet to C57BL/6J mice for 3 days. Similar to mice subjected to leucine deprivation for 7 days,^[^ ^31^ ^]^ those subjected to leucine deprivation for 3 days showed increased rectal temperature compared to those fed the control diet (Figure S1A, Supporting Information). Leucine deprivation led to higher resting state BAT surface temperature as measured by infrared thermal imager (Figure S1B, Supporting Information), and smaller lipid droplets in the BAT as shown by hematoxylin and eosin (H&E) staining, indicating higher lipid expenditure (Figure S1C, Supporting Information). To further confirm this phenomenon, we used reverse transcription polymerase chain reaction (RT‐PCR) and western blot analysis to examine a key gene involved in thermogenesis, uncoupling protein‐1 (UCP1) in BAT.^[^ ^31^ ^]^ We found that UCP1 expression was increased in leucine‐deprived mice (Figure S1D,E, Supporting Information). In addition, the expression of tyrosine hydroxylase (TH), implying the innervation of sympathetic nervous system in BAT,^[^ ^32^ ^]^ was increased after leucine deprivation (Figure S1E, Supporting Information). In order to detect sympathetic activation, norepinephrine (NE) turnover was performed in mice fed a control or leucine‐deficient diet following intraperitoneally (i.p.) injected with α‐methyl‐p‐tyrosine (α‐MPT) or saline.^[^ ^33^ ^]^ The basal NE levels in leucine‐deficient mice were significantly higher, but decreased more significantly 2 h post‐injection of α‐MPT compared with the control mice (Figure S1F, Supporting Information). This finding indicates a higher NE turnover in leucine‐deficient mice. The activation of sympathetic nervous system was further confirmed by the increased levels of cyclic adenosine monophosphate (cAMP) and phosphorylated protein kinase A (p‐PKA) substrates (Figure S1G,H, Supporting Information), downstream signals from β‐adrenergic receptor pathway.^[^ ^34^ ^]^ Moreover, tissue weight of subcutaneous white adipose tissue (sWAT), epididymal white adipose tissue (eWAT), and BAT were reduced after leucine deprivation (Figure S1I, Supporting Information). Consistently, liver mass and gastrocnemius muscle mass also decreased as leucine deprivation promotes autophagy and accelerates protein degradation (Figure S1J,K, Supporting Information).^[^ ^35^ ^]^ Meanwhile, leucine deficiency leads to an increase in oxygen consumption without altering locomotor activity (Figure S1L,M, Supporting Information), as shown previously.^[^ ^29^, ^36^ ^]^ These data confirm that 3 days of leucine deprivation can enhance BAT thermogenesis in mice, leading to increased body temperature and energy expenditure.

**Figure 1:**
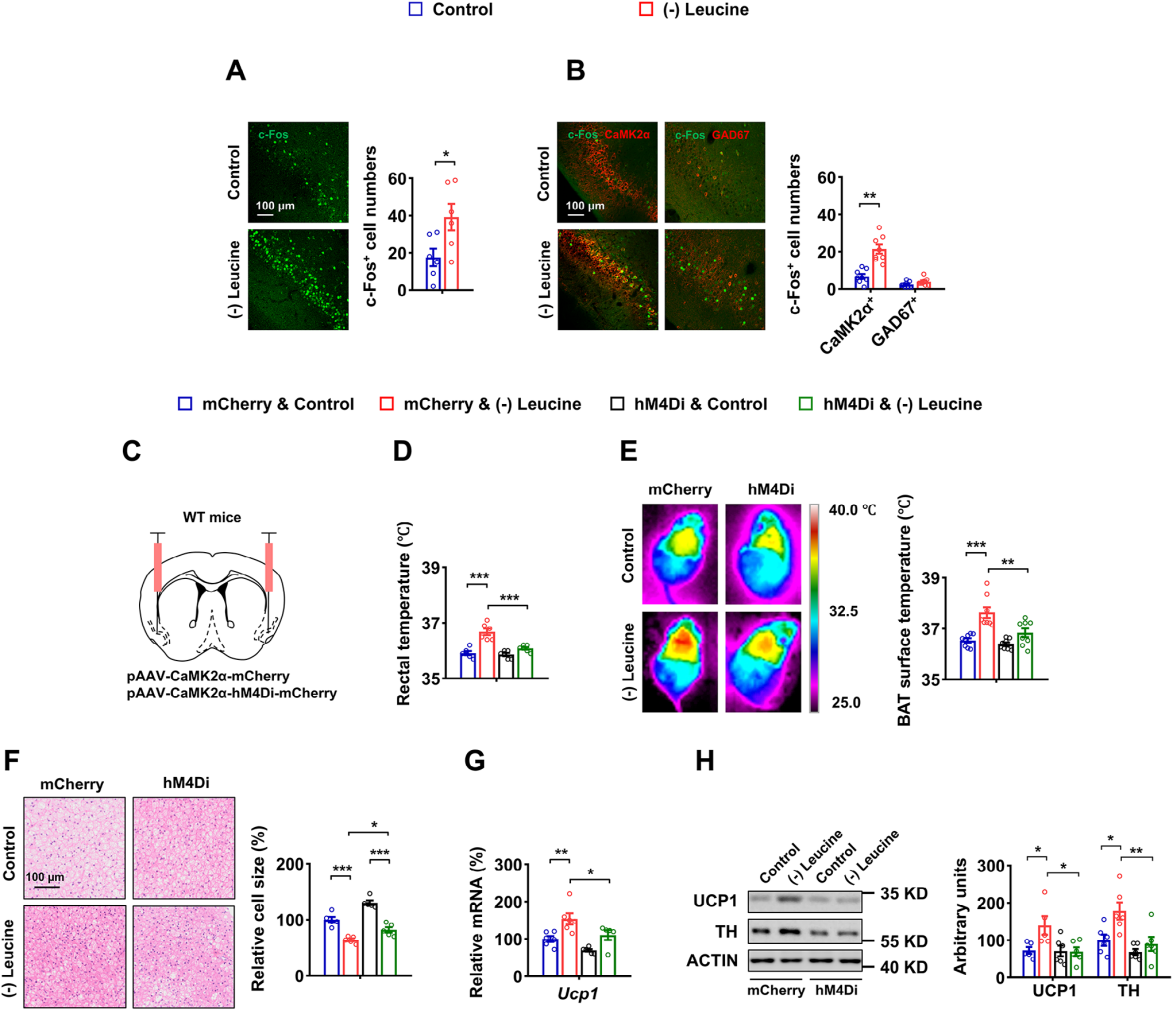
Silencing APC glutamatergic neurons blocks BAT thermogenesis under leucine deprivation. A, B) 8‐ to 12‐week‐old male wild‐type (WT) mice fed a control (Control) or leucine‐deficient [(–) Leucine] diet for 3 days. A) Immunofluorescence (IF) staining for c‐Fos (green) in anterior piriform cortex (APC) sections (left), and quantification of c‐Fos positive cell numbers (right, n = 6 per group). B) IF staining for CaMK2α or GAD67 (red), c‐Fos (green), and merge (yellow) in APC sections (left), and quantification of c‐Fos and CaMK2α or GAD67 colocalized cell numbers (right, n = 8 per group). C–H) 8‐ to 12‐week‐old male WT mice receiving AAVs expressing CaMK2α‐mCherry (mCherry) or CaMK2α‐hM4Di (hM4Di), all received CNO injections every 12 h for 3 days, simultaneously fed a Control or (–) Leucine diet for 3 days. C) Schematics of virus‐mediated hM4Di expression (red) sites. D) Rectal temperature (n = 6 per group). E) Representative infrared thermal images (left) and the quantifications (right, n = 8 per group). F) Representative images of hematoxylin and eosin (H&E) staining of BAT (left) and BAT cell size quantified by ImageJ analysis of H&E images (right, n = 5 per group). G) *Ucp1* mRNA in BAT (n = 5–6 per group). H) UCP1 and TH protein levels in BAT (left) and quantified by densitometric analysis (right, n = 5–6 per group). Data are represented as mean ± SEM. Statistical analyses were performed by two‐tailed unpaired Student's t test for (B), or by two‐way ANOVA with Tukey's multiple comparisons test for (C–H); *p < 0.05, **p < 0.01, and ***p < 0.001.

To investigate whether the APC brain region responds to leucine deprivation, we examined the protein levels of the immediate early gene c‐Fos, a marker of activated neurons,^[^ ^36^ ^]^ in mice after 3 days of leucine deprivation using immunofluorescence (IF) staining. The number of c‐Fos‐positive neurons increased in APC of mice subjected to leucine deprivation (**Figure** **1**A).

To gain genetic access to leucine‐sensing neurons within the APC, we searched for cell‐type markers co‐expressed with c‐Fos in leucine‐deprived mice. CaMK2α is a marker for glutamatergic neurons in the forebrain, while GAD67 marks GABAergic neurons.^[^ ^37^, ^38^ ^]^ Notably, glutamatergic neurons exhibited a significant increase in c‐Fos signaling following leucine deprivation, whereas GABAergic neurons displayed no difference (Figure 1B). The findings suggest that the leucine‐sensing neurons are confined to a range of excitatory glutamatergic neurons.

To confirm whether these neurons are involved in regulating BAT thermogenesis, designer receptors exclusively activated by designer drugs (DREADD) experiments were conducted. Adeno‐associated viruses (AAVs) encoding hM4Di (AAV‐CaMK2α‐hM4Di mCherry) or mCherry alone (AAV‐CaMK2α‐mCherry) under CaMK2α promoter control were bilaterally injected into the APC of wild‐type (WT) male mice (Figure 1C). All mice were i.p. injected with the DREADD agonist clozapine‐N‐oxide (CNO) 4 weeks after AAV delivery. CNO injection inhibited c‐Fos expression in APC glutamatergic cells labelled with mCherry, which marks hM4Di^+^ neurons (Figure S2A, Supporting Information). Inhibiting the neuronal activity of APC glutamatergic neurons largely reversed leucine deprivation‐induced BAT thermogenesis, as demonstrated by changes in rectal temperature, BAT surface temperature, BAT cell size, UCP1 expression, TH protein (Figure 1D–H) and other related parameters (Figure S2B,C, Supporting Information). These data suggest that APC glutamatergic neurons are essential for the BAT thermogenesis induced by leucine deprivation and may act through the sympathetic nervous system. APC has been previously documented to play a role in the aversion response to a diet deficient in essential amino acids.^[^ ^22^ ^]^ Consistently, our results demonstrated that inhibition of APC glutamatergic neurons reversed the inhibitory effect of leucine deprivation on food intake and energy intake for the first 12 h, with no significant effect thereafter (Figure S2D,E, Supporting Information).

### 2.2 APC^CRH^ Neurons Regulate BAT Thermogenesis under Leucine Deprivation

**Figure 2:**
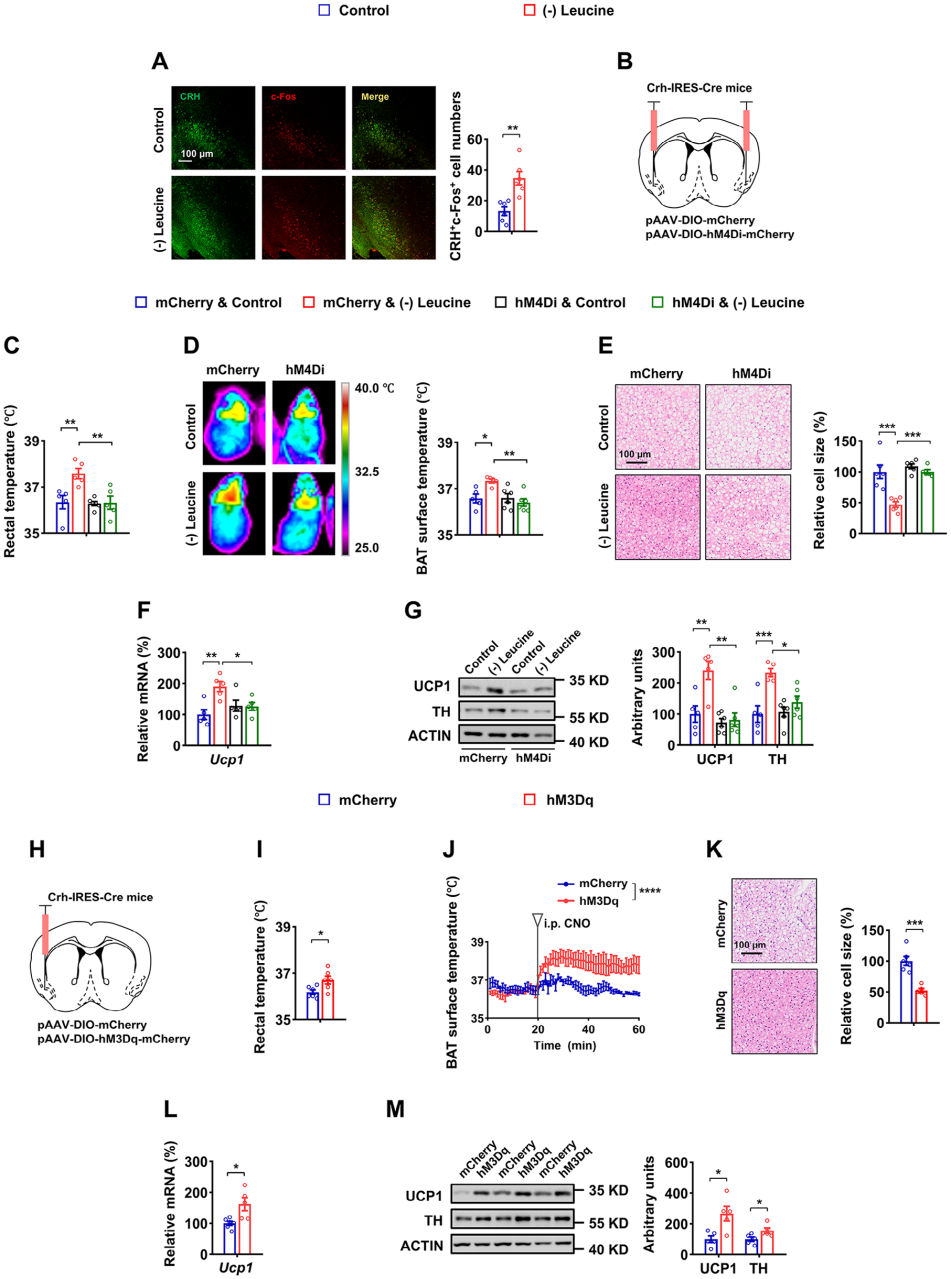
APC^CRH^ neurons play an essential role in regulating BAT thermogenesis. A) 8‐ to 12‐week‐old male WT mice fed Control or (–) Leucine diet for 3 days.IF staining for corticotropin‐releasing hormone (CRH) (green), c‐Fos (red), and merge (yellow) in APC sections (left), and quantification of CRH and c‐Fos colocalized cell numbers (right, n = 6 per group). B‐G) 8‐ to 12‐week‐old male Crh‐IRES‐Cre mice receiving AAVs expressing DIO‐mCherry (mCherry) or DIO‐hM4Di (hM4Di), all received CNO injections every 12 h for 3 days, simultaneously fed a Control or (–) Leucine diet for 3 days. B) Schematics of virus‐mediated hM4Di expression (red) sites. C) Rectal temperature (n = 5 per group). D) Representative infrared thermal images (left) and the quantifications (right, n = 5–6 per group). E) Representative images of H&E staining of BAT (left) and BAT cell size quantified by Image J analysis of H&E images (right, n = 5–6 per group). F) *Ucp1* mRNA in BAT (n = 5 per group). G) UCP1 and TH protein levels in BAT (left) and quantified by densitometric analysis (right, n = 5–6 per group). H–M) 8‐ to 12‐week‐old male Crh‐IRES‐Cre mice receiving AAVs expressing DIO‐mCherry (mCherry) or DIO‐hM3Dq (hM3Dq), all received CNO injections every 12 h for 3 days, simultaneously fed a normal chow diet. H) Schematics of virus‐mediated hM3Dq expression (red) sites. I)Rectal temperature (n = 6 per group). J) Representative infrared thermal images (left) and quantifications (right, n = 5 per group). K) Representative images of H&E staining of BAT (left) and quantifications (right, n = 5–6 per group). L) *Ucp1* mRNA in BAT (n = 5–6 per group). M) UCP1 and TH protein levels in BAT (left) and quantifications (right, n = 5 per group). Data are represented as mean ± SEM. Statistical analyses were performed by two‐tailed unpaired Student's t test for (A, I–M), or by two‐way ANOVA with Tukey's multiple comparisons test for (C–G); *p < 0.05, **p < 0.01, ***p < 0.001, and ****p < 0.0001.

To further characterize APC cell types that are sensitive to leucine fluctuations, we analyzed a subset of glutamatergic neurons in the APC. According to the Allen Brain Atlas database, CRH neurons are abundant in the APC (Figure S3A, Supporting Information).^[^ ^39^ ^]^ However, their functional roles in the APC remain unreported. As hypothalamic CRH neurons play a crucial role in the composition of the central stress system,^[^ ^40^ ^]^ and leucine deprivation may serve as a potential nutritional stress signal, it is imperative to investigate whether leucine deprivation can activate APC^CRH^ neurons. Through IF staining, we discovered that 3 days of leucine deprivation increased c‐Fos signaling in APC^CRH^ neurons (**Figure** **2**A), suggesting that leucine deprivation activates these neurons.

After establishing the involvement of APC^CRH^ neurons in leucine sensing, we investigated their specific roles in thermoregulation. To this end, a Cre‐dependent AAV encoding hM4Di (AAV‐DIO‐hM4Di‐mCherry) or mCherry (AAV‐DIO‐mCherry) was bilaterally injected into the APC of Crh‐IRES‐Cre mice (Figure 2B). All of these mice were then i.p. injected with CNO 4 weeks after AAV delivery. The inhibition of APC^CRH^ neuronal activity was confirmed by reduced IF staining of c‐Fos in the APC^CRH^ neurons (reflected by mCherry) of mice injected with AAV‐hM4Di (Figure S3B, Supporting Information). Inhibition of APC^CRH^ neurons blocked the effect of leucine deprivation‐induced BAT thermogenesis, as demonstrated by corresponding changes in rectal temperature, BAT surface temperature, BAT cell size, UCP1 and TH expression, NE levels, and fat mass (Figure 2C–G; Figure S3C,D, Supporting Information). This suggests that APC^CRH^ neuronal activation is essential for leucine deficiency to enhance BAT thermogenesis.

Next, we examined whether the activation of APC^CRH^ neurons was adequate for BAT thermogenesis under normal chow‐diet conditions. We unilaterally delivered AAV‐DIO‐hM3Dq‐mCherry or control AAV to the APC of Crh‐IRES‐Cre mice to activate APC^CRH^ neurons (Figure 2H; Figure S4A, Supporting Information). After recovery of the mice, i.p. administration of CNO for 3 days resulted in a significant increase in rectal and BAT surface temperature, as well as a notable reduction in BAT cell size (Figure 2I–K). These changes were accompanied by alterations in BAT UCP1 and TH expression levels (Figure 2L,M), as well as in NE levels and fat mass (Figure S4B,C, Supporting Information), indicating that the activation of APC^CRH^ neurons resembles leucine deprivation. Furthermore, APC^CRH^ neuron activation reduced food intake and energy intake (Figure S4D,E, Supporting Information). Our results suggest that APC^CRH^ neurons play a crucial regulatory role in BAT thermogenesis.

### 2.3 APC^CRH^ Neurons Increase Intrinsic Excitability through GCN2

**Figure 3:**
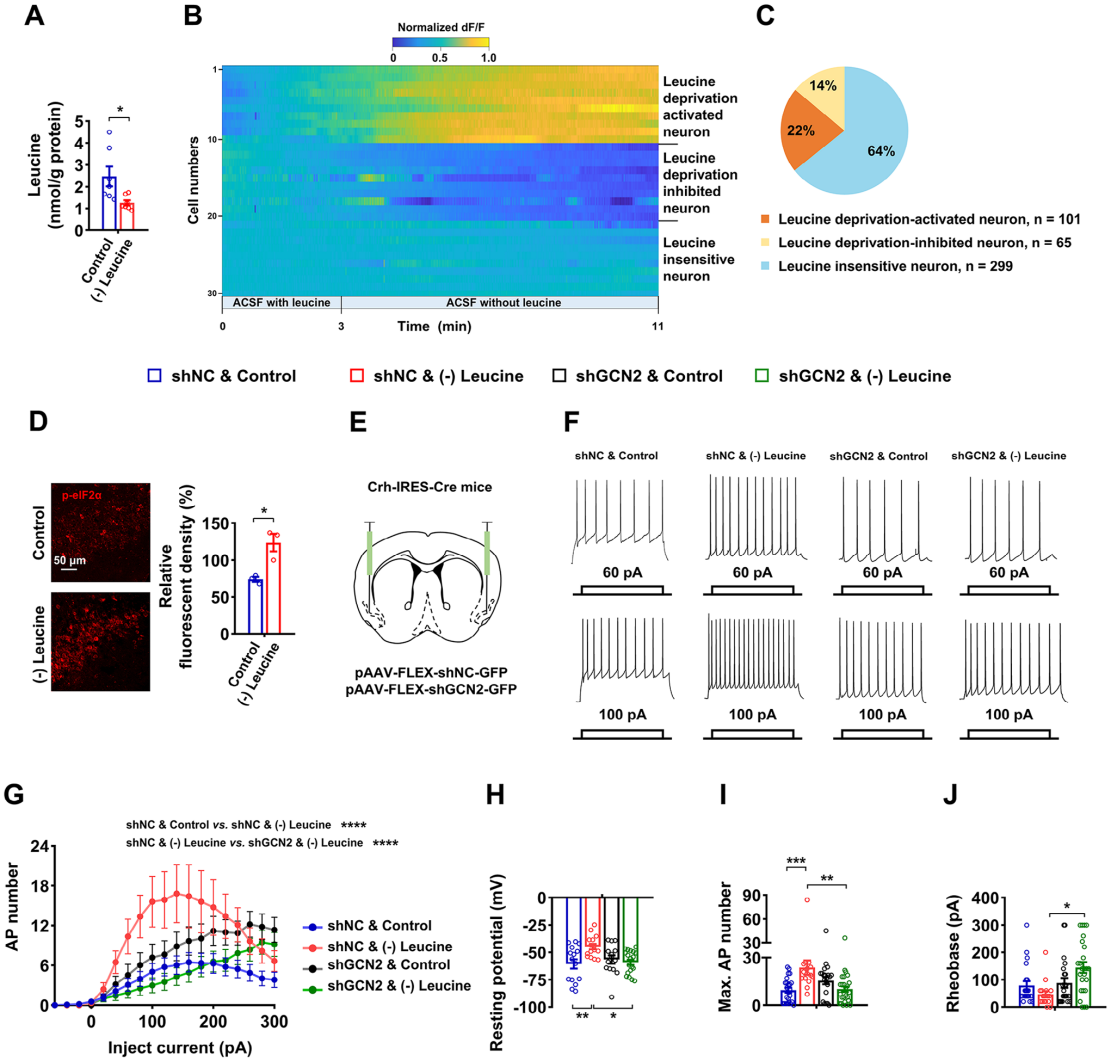
APC^CRH^ neurons increase intrinsic excitability through GCN2. A, D) 8‐ to 12‐week‐old male WT mice fed Control or (–) Leucine diet for 3 days. A) Leucine levels in the APC (n = 7 per group). B, C) 8‐ to 12‐week‐old male Crh‐IRES‐Cre mice receiving AAVs expressing DIO‐jGCaMP7s, APC brain slices were isolated and promptly incubated in an artificial cerebrospinal fluid (ACSF) with 100 µM leucine, followed by amino acid‐free ACSF. B) Representative leucine deprivation‐induced calcium responses. Rows: dF/F from ImageJ‐detected events per cell, normalized between 0 and 1 per cell (n = 10 per group). C) Quantifications of different responses to leucine deprivation of APC^CRH^ neurons. D) IF staining for phosphorylation at serine 51 site of eukaryotic translation initiation factor 2 subunit 1 (p‐eIF2α) (red) in APC sections (left), and quantifications (right, n = 3 per group). E–J) 8‐ to 12‐week‐old male WT mice receiving AAVs expressing FLEX‐shNC‐GFP (shNC) or FLEX‐shGCN2‐GFP (shGCN2), all fed a Control or (–) Leucine diet for 3 days. E) Schematics of virus‐mediated shGCN2 expression (green) sites. F) Representative current‐clamp recording traces of APC^CRH^ cells with 60 and 100 pA current injections. G) F‐I curve of APC^CRH^ cells (n = 17–25 per group). H) Resting membrane (n = 15–19 per group). I) Maximum action potential number across current steps (n = 15–25 per group). J) Rheobase of APC^CRH^ cells (n = 17–25 per group). Data are represented as mean ± SEM. Statistical analyses were performed by two‐tailed unpaired Student's t test for (A, D), or by two‐way ANOVA with Tukey's multiple comparisons test for (F–J); *p < 0.05, **p < 0.01, **p < 0.001, and ****p < 0.0001.

Consistent with prior studies demonstrating that a leucine‐deficient diet for 3 days reduces serum leucine levels,^[^ ^36^ ^]^ our findings also indicate a decrease in leucine within the APC (**Figure** **3**A). We confirmed that some APC^CRH^ neurons were sensitive to leucine deprivation using calcium signaling recordings (Figure 3B). Approximately 22% of neurons exhibited activation in response to leucine deprivation (a subset we designated as leucine deprivation‐activated neurons), while about 14% of neurons showed inhibition under identical conditions (classified as leucine deprivation‐inhibited neurons), and the remaining 64% of neurons did not respond to changes in leucine levels (categorized as leucine insensitive neurons) (Figure 3C). To investigate the potential involvement of GCN2 in APC^CRH^ neurons sensing leucine deprivation, we examined p‐eIF2α levels in the APC of leucine‐deprived mice and observed an increase (Figure 3D). Given that GCN2 plays a crucial role in detecting amino acid insufficiency, we hypothesized that changes in APC^CRH^ neuronal activity are GCN2‐dependent. To this end, Cre‐dependent AAVs encoding short hairpin RNA directed against GCN2 (AAV‐FLEX‐shGCN2‐GFP) or GFP (AAV‐FLEX‐shNC‐GFP) were bilaterally injected into the APC of Crh‐IRES‐Cre mice (Figure 3E). Using p‐eIF2α, we demonstrated that GCN2 signaling in APC^CRH^ neurons was activated by leucine deprivation and that shGCN2 AAV reduced the GCN2 signaling induced by leucine deprivation (Figure S5A, Supporting Information). Following a 6‐week recovery period, the mice underwent leucine deprivation for 3 days, and the APC brain sections were promptly isolated for whole‐cell patch‐clamp recordings of GFP‐positive cells within the APC brain region (Figure S5B, Supporting Information). We then produced frequency–current (F–I) curves of the APC^CRH^ cells.^[^ ^41^ ^]^ Leucine deprivation increased the firing rate of neurons at multiple steps which was reversed by GCN2 knockdown, as represented by 60 pA and 100 pA sweeps (Figure 3F,G). Both resting membrane potential and maximum action potential (AP) number were significantly elevated due to leucine deprivation, which were reduced by GCN2 knockdown (Figure 3H,I). Rheobase showed a trend toward decrease in leucine deprivation, which was also reversed by GCN2 knockdown (Figure 3J). These data provide compelling evidence that leucine deprivation modulates the activity of leucine‐sensing APC^CRH^ neurons via the GCN2 pathway.

### 2.4 Knockdown of GCN2 in APC^CRH^ Neurons Blocks Leucine Deprivation‐Induced BAT Thermogenesis

**Figure 4:**
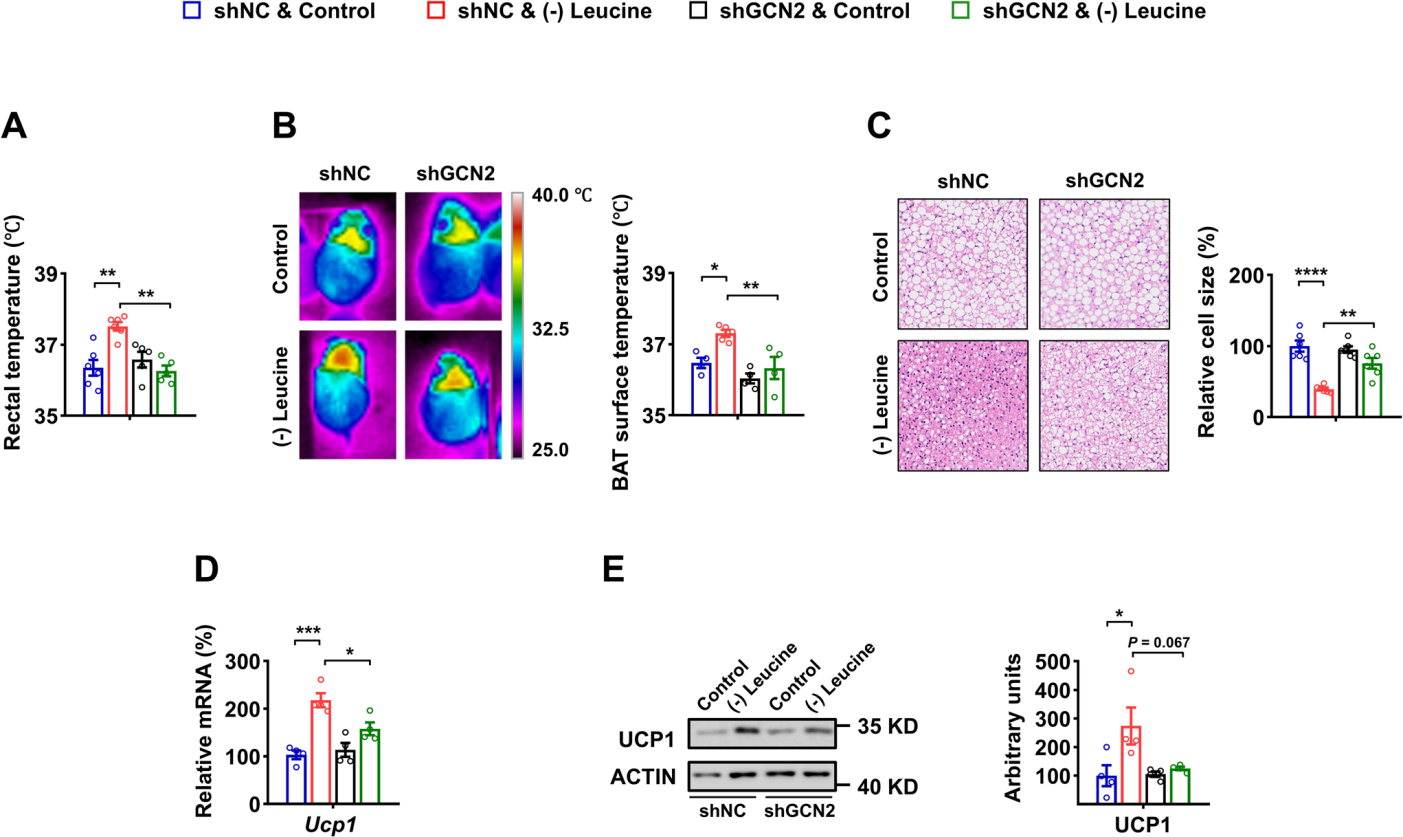
GCN2 in APC^CRH^ neurons is necessary for BAT thermogenesis induced by leucine deprivation. 8‐ to 12‐week‐old male WT mice receiving AAVs expressing FLEX‐shNC‐GFP (shNC) or FLEX‐shGCN2‐GFP (shGCN2), all fed a Control or (–) Leucine diet for 3 days. A) Rectal temperature (n = 5–6 per group). B) Representative infrared thermal images (left) and quantifications (right, n = 4–5 per group). C) Representative images of H&E staining of BAT (left) and quantifications (right, n = 6 per group). D) *Ucp1* mRNA in BAT (n = 4 per group). E) UCP1 protein levels in BAT (left) and quantifications (right, n = 4 per group). Data are represented as mean ± SEM. Statistical analyses were performed by two‐way ANOVA with Tukey's multiple comparisons test; *p < 0.05, **p < 0.01, ***p < 0.001, and **** *p* < 0.0001.

We then investigated whether the knockdown of GCN2 in APC^CRH^ neurons could block leucine deprivation‐induced BAT thermogenesis. As confirmed by examining the rectal temperature, BAT surface temperature, BAT cell size, and UCP1 protein expression in BAT, the suppression of GCN2 in APC^CRH^ neurons impeded the BAT thermogenesis induced by leucine deprivation (**Figure** **4**A–E). These findings provide additional evidence supporting the vital role of GCN2 in APC^CRH^ neurons to regulate the BAT thermogenesis induced by leucine deprivation.

Furthermore, if GCN2 activation in the APC was responsible for BAT thermogenesis under leucine deprivation, knockdown of APC GCN2 would be expected to exert a blocking effect. To test this hypothesis, we bilaterally injected AAVs expressing Cre‐GFP or GFP into the APC of GCN2 loxp/loxp mice, to knockdown GCN2 exclusively in the APC (GCN2 KD) or to produce the control (GCN2^+/+^), respectively (Figure S6A, Supporting Information). These mice were then maintained on a control or leucine‐deprived diet for 3 days. RT‐PCR analyses revealed that GCN2 levels were significantly decreased in the APC of GCN2 KD mice (Figure S6B, Supporting Information). We then determined the effect of APC GCN2‐knockdown blocked leucine deprivation‐induced BAT thermogenesis, as demonstrated by the corresponding changes in rectal temperature, BAT surface temperature, BAT cell size, UCP1 expression, TH expression and NE levels (Figure S6C–H, Supporting Information). Taken together, these data indicate that BAT thermogenesis in response to leucine deprivation requires GCN2 in the APC.

In addition, we investigated whether GCN2 expressed in APC‐glutamatergic neurons regulates BAT thermogenesis. We first identified GCN2 was highly expressed in APC glutamatergic neurons (Figure S7A, Supporting Information), we then knocked down GCN2 in these neurons (Figure S7B, Supporting Information). The changes in p‐eIF2α levels demonstrated a significant functional inhibition of GCN2 in the knockdown group (Figure S7C, Supporting Information). Our findings revealed that GCN2 knockdown in glutamatergic neurons inhibited the BAT thermogenesis induced by leucine deprivation, as demonstrated by the restoration of BAT rectal and surface temperature, UCP1 levels, and corresponding changes in related parameters (Figure S7D–J, Supporting Information). In addition, the increased c‐Fos expression in APC glutamatergic neurons under leucine deprivation was largely blunted by the knockdown of GCN2 (Figure S7K, Supporting Information). Taken together, APC GCN2 plays a crucial role in neuronal activation and the regulation of BAT thermogenesis induced by leucine deprivation.

### 2.5 The APC–LH Circuit Regulates BAT Thermogenesis under Leucine Deprivation

**Figure 5:**
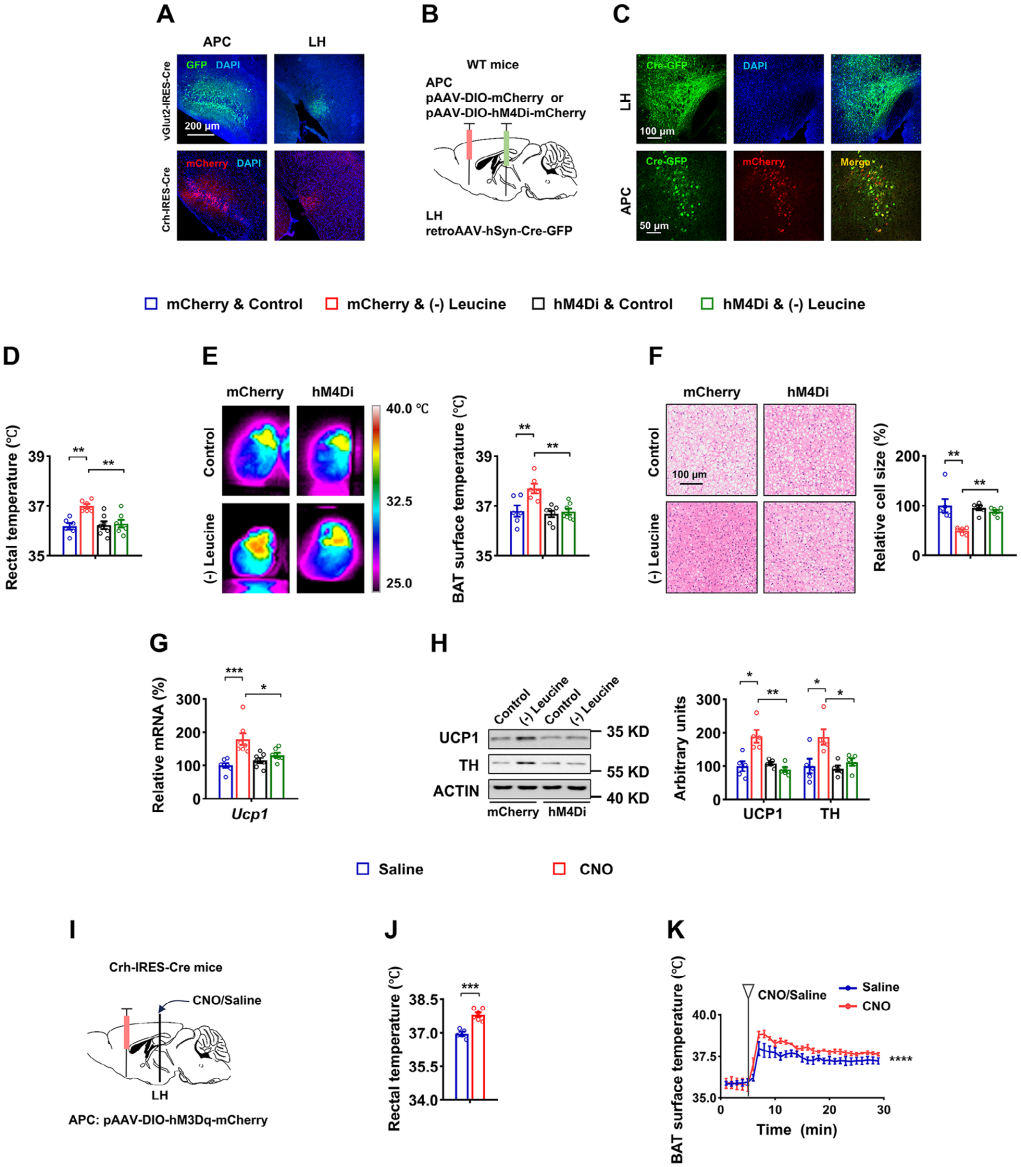
Inhibition of the APC–LH circuit blocks BAT thermogenesis under leucine deprivation. A) IF images of APC (left) and LH (right) sections labeled for GFP (green), mCherry (red), and DAPI (blue). B–H) 8‐ to 12‐week‐old male WT mice receiving retroAAV‐EGFP‐2A‐Cre AAV to the LH and Cre‐dependent AAV‐DIO‐hM4Di‐mCherry or control AAV to the APC. All received CNO injections every 12 h for 3 days, simultaneously fed a Control or (–) Leucine diet for 3 days. B) Schematics of virus‐mediated hM4Di expression (red) sites and Cre expression (green) sites. C) IF images for mCherry (red), GFP (green), DAPI (blue), and merge (yellow) in LH (up) and APC sections (bottom). D) Rectal temperature (n = 6–7 per group). E) Representative infrared thermal images (left) and quantifications (right, n = 6–7 per group). F) Representative images of H&E staining of BAT (left) and quantifications (right, n = 6 per group). G) *Ucp1* mRNA in BAT (n = 7 per group). H) UCP1 and TH protein levels in BAT (left) and quantifications (right, n = 5 per group). J–K) 8‐ to 12‐week‐old male Crh‐IRES‐Cre mice receiving AAVs expressing DIO‐hM3Dq (hM3Dq) in APC and guide cannulas implanting in LH (with saline or CNO injecting to cannulas). I) Schematic of virus injection. J) Rectal temperature (n = 5–6 per group). K) BAT surface temperature (n = 5–6 per group). Data are represented as mean ± SEM. Statistical analyses were performed by two‐tailed unpaired Student's t test for (J–K) or two‐way ANOVA with Tukey's multiple comparisons test for (D–H); *p < 0.05, **p < 0.01, **p < 0.001, and ***p < 0.0001.

Many of the classical brain regions involved in thermoregulation have been previously documented.^[^ ^42^, ^43^, ^44^, ^45^ ^]^ APC^CRH^ neurons may function in a coordinated manner in these regions to regulate BAT thermogenesis. To map possible neural circuits, we injected AAV‐DIO‐GFP into the APC of vGlut2‐IRES‐Cre mice and AAV‐DIO‐mCherry into the APC of Crh‐IRES‐Cre mice to visualize axonal projections. GFP^+^ and mCherry^+^ fibers were observed in the LH (**Figure** **5**A), indicating that APC^CRH^ neurons project into the LH. The LH has long been implicated in the control of BAT thermogenesis. However, the mechanism underlying its input remains unclear. To investigate the involvement of the APC–LH circuit in the regulation of leucine deprivation‐induced BAT thermogenesis, we first tested the necessity of APC‐to‐LH projections for BAT thermoregulation. We targeted hM4Di to the APC neurons innervating the LH by delivering retroAAV‐EGFP‐2A‐Cre AAV to the LH and Cre‐dependent AAV‐DIO‐hM4Di‐mCherry or AAV‐DIO‐mCherry to the APC of WT mice (Figure 5B,C, Figure S8A, Supporting Information).^[^ ^46^ ^]^ We found that the inhibition of LH‐projecting APC neurons by CNO significantly attenuated the BAT thermogenesis induced by leucine deprivation, as demonstrated by corresponding changes in rectal temperature, BAT surface temperature, BAT cell size, fat mass, UCP1 and sympathetic signals (Figure 5D–H; Figure S8B,C, Supporting Information). These data suggest that the APC regulates thermogenesis via a circuit that projects into the LH.

Moreover, we activated the axon terminals of APC^CRH^ neurons that projected into the LH to determine whether this CRH‐to‐LH projection was sufficient for BAT thermoregulation. We injected Cre‐dependent hM3Dq‐mCherry AAV into the APC of Crh‐IRES‐Cre mice and locally delivered CNO or saline into the LH (Figure 5I).^[^ ^47^ ^]^ Higher rectal and BAT surface temperature were observed in the CNO group than in the saline group (Figure 5J,K). These data reveal that activation of the APC^CRH^–LH circuit is sufficient for BAT thermogenesis under a normal chow diet.

Taken together, these data suggest that the APC^CRH^–LH circuit is essential for the BAT thermogenesis induced by leucine deprivation.

### 2.6 APC^CRH^ Neurons Support Temperature Maintenance under Cold Exposure

**Figure 6:**
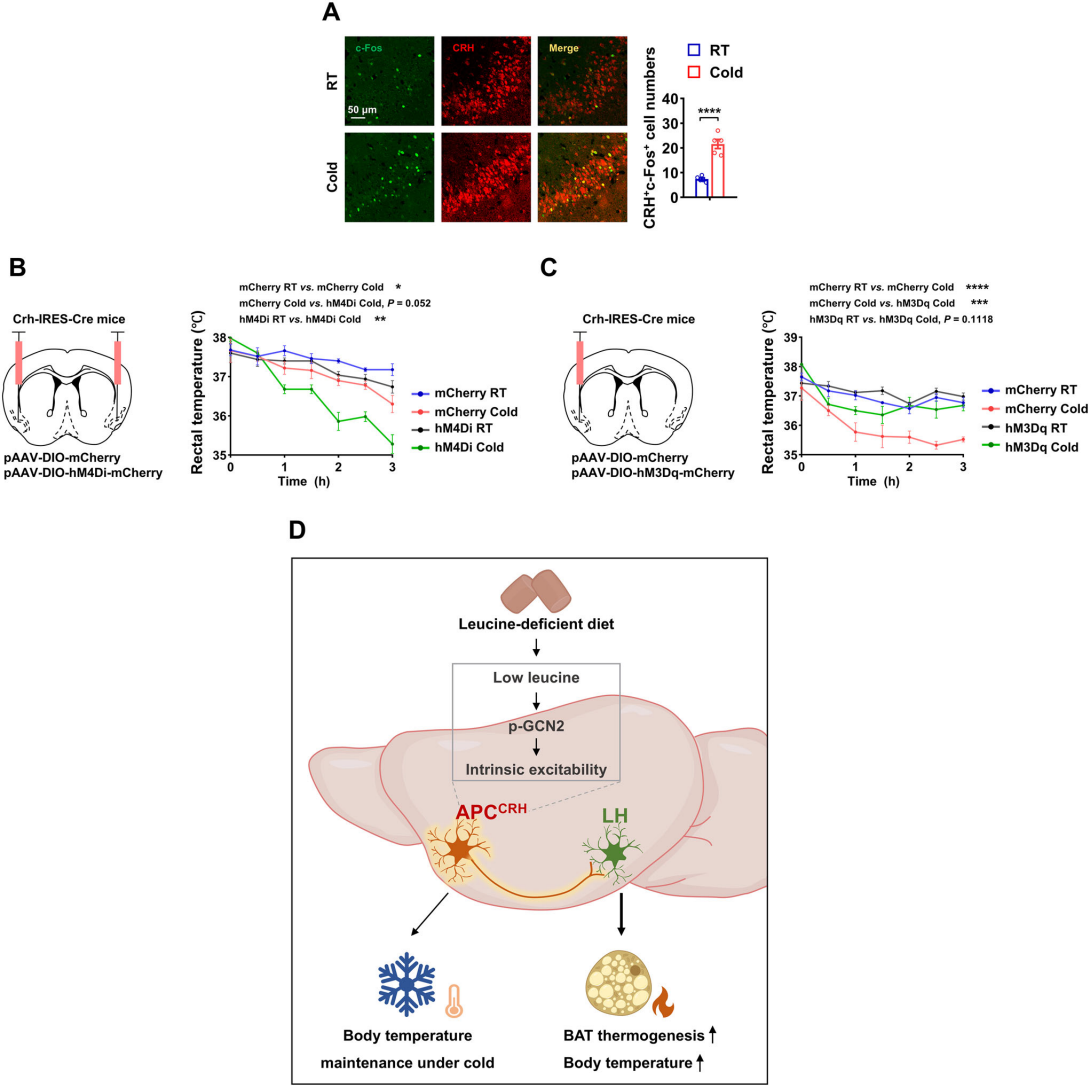
APC^CRH^ neurons support cold‐related temperature maintenance. A) 8‐ to 12‐week‐old male WT mice at 25 °C (RT) or 4 °C (Cold) for 3 h in the absence of food and water. A) IF staining for c‐Fos (green), CRH (red), and merge (yellow) in APC sections (left), and quantifications (right, n = 5 per group). B) 8‐ to 12‐week‐old male Crh‐IRES‐Cre mice receiving AAVs expressing DIO‐mCherry (mCherry) or DIO‐hM4Di (hM4Di) at 25 °C (RT) or 4 °C (Cold) for 3 h in the absence of food and water. Schematics of virus‐mediated hM4Di expression (red) sites (left) and rectal temperature (right, n = 5 per group). C) 8‐ to 12‐week‐old male Crh‐IRES‐Cre mice receiving AAVs expressing DIO‐mCherry (mCherry) or DIO‐hM3Dq (hM3Dq) at 25 °C (RT) or 4 °C (Cold) for 3 h in the absence of food and water. Schematics of virus‐mediated hM3Dq expression (red) sites (left) and rectal temperature (right, n = 4–5 per group). D) Working model. Data are represented as mean ± SEM. Statistical analyses were performed by two‐tailed unpaired Student's t test for (A) or two‐way ANOVA with Tukey's multiple comparisons test for (B, C); *p < 0.05, **p < 0.01, ***p < 0.001, and ****p < 0.0001.

The discovery of the regulatory role of APC^CRH^ neurons in BAT thermogenesis induced by leucine deprivation prompted us to investigate their potential involvement in other physiological functions related to peripheral thermogenesis. Shivering thermogenesis plays a crucial role during cold exposure, and non‐shivering thermogenesis, which mainly occurs in BAT, is also highly significant.^[^ ^43^ ^]^ Given that BAT thermogenesis contributes to body temperature maintenance under cold conditions,^[^ ^48^, ^49^ ^]^ we examined whether mouse APC^CRH^ neurons were responsive to cold stimulation. Upon exposure to cold, APC^CRH^ neuron activity increased, as evidenced by a higher number of c‐Fos‐positive neurons (**Figure** **6**A). In the case of acute cold exposure, mice exhibit a decrease in body temperature, with slight differences in degree among different individuals.^[^ ^50^, ^51^, ^52^ ^]^ Therefore, we investigated the involvement of APC^CRH^ neurons in this process. Mice with or without chemogenetically suppressed or activated APC^CRH^ neuron activity were subjected to either cold or room‐temperature conditions (25 °C), and changes in rectal temperature were monitored (Figure 6B,C). While control mice decreased rectal temperature under cold conditions, mice with inhibited APC^CRH^ neuronal activity exhibited a more severe decrease in rectal temperature (Figure 6B). Furthermore, neuronal inhibition did not affect the basal body temperature of the mice at 25 °C (Figure 6B). Conversely, chemogenetically activated APC^CRH^ neurons maintained relative stable body temperature during 3‐h cold exposure (Figure 6C). These findings suggest that APC^CRH^ neurons play a significant role in regulating body temperature during exposure to cold stimuli.

## 3 Discussion

In the present study, we identified a population of leucine‐sensing CRH neurons in the APC that senses leucine deprivation and regulates BAT thermogenesis. Moreover, these neurons response to nutrient and temperature signals to maintain body temperature homeostasis and modulate energy metabolism (Figure 6D).

Identifying specific nutrient‐sensing neurons helps us understand the neurocircuit‐related regulation of metabolic diseases. Glucose‐sensitive neurons are the most well‐studied nutrient‐sensing neurons,^[^ ^8^ ^]^ while little is known about specific amino acid‐ sensing neurons. This has become an increasingly important issue, especially as research on the importance of amino acids in metabolic diseases progresses. In this study, we identified a class of neurons in the APC that senses amino acids.

We focused on the APC brain region for two reasons. First, it is known to be an amino acid‐sensing region, but the specific types of neurons that sense amino acids remain inadequately comprehended. Second, the APC reportedly participates in an aversive response during amino acid deprivation, and the additional metabolic effects are poorly understood and require further elucidation. The highest concentration of CRH neurons is found in the hypothalamic paraventricular nucleus, which mediates the stress response.^[^ ^53^ ^]^ In addition, the CRH peptide is present at several extra‐hypothalamic sites in the brain, most of which are related to the limbic system and stress circuitry.^[^ ^54^ ^]^ Though APC also contains a substantial population of CRH neurons, their functions have not yet been elucidated.

Further, we demonstrated the important role of APC^CRH^ neurons in BAT thermogenesis and body temperature regulation under essential amino acid deprivation. Our study elucidates the previously undocumented functions of CRH neurons in the APC. Moreover, the APC constitutes a segment of the olfactory cortex primarily engaged in encoding odorous information,^[^ ^55^, ^56^ ^]^ which has seldom been associated with thermogenesis or the regulation of body temperature. Thus, our findings demonstrate a thermoregulation function of the olfactory cortex that has not been previously reported.

Leucine‐deficient diet led to fat loss and metabolic improvement. The decreased energy intake and increased energy expenditure, such as enhanced lipolysis and thermogenesis, may have contributed to these effects.^[^ ^29^ ^]^ In this study, we identified a thermogenic regulatory function of APC. The body is expected to reduce energy expenditure in response to chronic energy shortages caused by the loss of essential amino acids. However, it is intriguing that the body continues to produce heat under leucine‐deprived conditions. We propose that leucine deprivation serves as a nutritional stress signal that promotes a nutritional stress response designed to enhance survival by motivating mice to seek more nutritionally comprehensive food sources. The central limbic system regulates stress‐induced thermogenesis and augments physical and neural performance by raising muscle and central nervous system temperature by a few degrees Celsius, which is advantageous for survival during fight or flight situations when animals encounter threats.^[^ ^57^, ^58^, ^59^ ^]^ The APC is part of the central limbic system, and has been demonstrated to be activated by predator odor, a high‐salt diet, restraint, and other stresses.^[^ ^60^ ^]^ Here, we speculate that the APC detects nutrient stress and coordinates thermogenesis to facilitate thermoregulation as part of the nutritional stress response.

Also, we explored the signaling pathways that affect CRH neuronal activity. It is widely recognized that GCN2 functions as an amino acid sensor and plays a crucial role in adaptive responses across various conditions.^[^ ^28^, ^61^ ^]^ In the central nervous system, GCN2 also contributes to the regulation of circadian rhythms.^[^ ^27^ ^]^ Given its high expression in the APC, we speculated that it might play an important role in metabolic control under leucine deprivation. This was confirmed by knocking down of GCN2 expression in both the APC brain region and specific neurons. This study provides evidence for the activation of GCN2 in APC^CRH^ neurons and highlights its involvement in leucine deprivation‐induced BAT thermogenesis. Additionally, our findings suggest that the regulatory effects exerted by APC^CRH^ GCN2 on thermogenesis is mediated by the stimulation of intrinsic neural properties, suggesting that GCN2 is involved in the modulation of neuronal activity. Furthermore, the specific pathway through which GCN2 alters neuronal activity has not yet been elucidated, and additional research is required to investigate the downstream targets of GCN2 that modulate intrinsic excitability.

We further explored the possible neural circuits directed by APC^CRH^ neurons. The APC projects into various brain regions, which may mediate its effects on thermogenic regulation.^[^ ^62^, ^63^, ^64^ ^]^ Our findings demonstrate that APC^CRH^ neurons project into the LH. The LH is a well‐established site for the regulation of BAT thermogenesis, integrating synaptic inputs from diverse brain regions and cell types to modulate food intake and energy expenditure.^[^ ^65^, ^66^ ^]^ Extra‐hypothalamic limbic centers feature prominently among the LH afferents that mediate energy balance, but their contributions to energy balance remain unclear.^[^ ^67^ ^]^ The circuit formed by APC‐to‐LH projections mediates leucine deprivation‐induced BAT thermogenesis, thereby enhancing our understanding of the neural networks involved in afferent signaling toward the hypothalamus. Nevertheless, our observations indicate that APC^CRH^ neurons also project into other brain regions, including the thalamus (Data not shown). Therefore, further studies are warranted to investigate and rule out the potential roles of these circuits.

In light of the pivotal role that BAT thermogenesis plays in the maintenance of body temperature during cold exposure, coupled with our discovery that APC^CRH^ neurons regulate BAT thermogenesis, we sought to investigate the potential involvement of APC^CRH^ neurons in the modulation of body temperature under cold conditions. Our empirical findings unequivocally demonstrate that APC^CRH^ neurons are activated by cold, and the activity of APC^CRH^ neurons significantly influences the maintenance of body temperature. It is important to emphasize, however, that both shivering thermogenesis and non‐shivering thermogenesis are essential components in the preservation of body temperature in response to cold stimuli. Consequently, a more in‐depth exploration is warranted to elucidate the precise molecular and physiological mechanisms through which APC^CRH^ neurons contribute to the regulation of body temperature, thereby advancing our understanding of this critical physiological process. Although our study suggests that APC^CRH^ neurons are required for body temperature control during cold exposure, the precise mechanisms by which cold signaling activates CRH neurons remain unclear. Furthermore, the relationship between amino acids and cold signaling has not been fully elucidated. Future studies should explore whether cold signaling activates CRH neurons via amino acid signaling. Alternatively, different subtypes of CRH neurons may exist in response to various stimuli.

However, several issues remain unanswered in the current study. It remains unclear why BAT thermogenesis is induced under leucine deprivation. Diet‐induced thermogenesis (DIT) might be one of the possibilities as reported under condition of protein deficiency.^[^ ^68^, ^69^, ^70^ ^]^ We are also uncertain about the mechanisms explaining the coexistence of the increase in BAT thermogenesis and the reduction in food intake. In addition to aversive response, it could result from an increase in DIT that generates a satiety signal to inhibit food intake, as observed in leucine‐deprived mice.^[^ ^71^, ^72^ ^]^ Alternatively, the occurrence of the increased BAT thermogenesis and reduced food intake can be caused by certain stress, as reported in other studies.^[^ ^59^, ^73^ ^]^ Nevertheless, additional validation of these hypotheses will be required in future studies.

In summary, this study dissects the properties and functions of APC^CRH^ neurons, demonstrating that they constitute a class of leucine‐sensing neurons and regulate BAT thermogenesis, therefore impacting body temperature and fat mass. The activation of these leucine‐sensing APC^CRH^ neurons is mediated by GCN2, and their thermogenic function is facilitated by a neural circuit projecting into the LH. Moreover, these neurons play an essential role in maintaining body temperature under cold conditions. Many important roles of amino acids in physiological processes and diseases have been identified, we anticipate that our findings will offer potential neural approaches for understanding metabolic control related to amino acids under various conditions, and may shed light on potential therapeutic targets to metabolic disorders.

## 4 Experimental Section

### Animals and Treatments

All experiments involving mice were approved in accordance with the guidelines of the Institutional Animal Care and Use Committee at Fudan University (ethical committee approval no. 2022030006S) and the Shanghai Institute of Nutrition and Health, Chinese Academy of Sciences (ethical committee approval no. SINH‐2020‐GFF‐1, SINH‐2021‐GFF‐1, and SINH‐2022‐GFF‐1). All male mice were C57BL/6J. GCN2‐floxed (GCN2^+/+^) mice were obtained from the Jackson Laboratory (Bar Harbor, ME, USA). The vGlut2‐IRES‐Cre mice were kindly provided by Prof. Yangang Sun from the Institute of Neuroscience, Chinese Academy of Sciences, while the Crh‐IRES‐Cre mice were kindly provided by Prof. Shumin Duan from the Institute for Translational Brain Research, Fudan University. The mice were kept under a controlled 12‐hour light/dark cycle, starting at 7 AM and ending at 7 PM, at a room temperature of 25°C. They had unrestricted access to a standard rodent diet (provided by Shanghai Pu Lu Teng Biotechnology, P1103F) and water. Euthanasia of the mice was performed via CO_2_ asphyxiation. For the studies involving acute cold exposure, each mouse was individually housed in a cage and exposed to a temperature of 4 °C without access to food, water, or bedding. In contrast, the control mice were subjected to the same conditions but maintained at a temperature of 25 °C. Control (nutritionally complete in terms of amino acids) and leucine‐deficient ((−) Leucine) diets were obtained from Trophic Animal Feed High‐Tech Co., Ltd (Nantong, China). All diets were isocaloric (Table S1, Supporting Information). For the feeding experiments, mice were acclimated to a control diet for 3 days and then randomly divided into control and (−) Leucine diet groups, with free access to their specified diet for 3 days, or as indicated. To evaluate the metabolic phenotypes resulting from a 3‐day leucine deprivation, WT mice were administered either a control diet or a leucine‐deficient diet for a duration of 3 days. Their food intake, body weight, fat mass, and lean mass were assessed both before the initiation and continuously throughout the entire 3‐day period.

### Stereotaxic Surgery and Viral Injections

Surgery was performed using a stereotaxic frame (RWD Life Science, Shenzhen, China). Throughout the procedure, the animals' body temperature was regulated with the aid of a heating pad. To ensure ocular lubrication, ophthalmic ointment was applied. The viruses were administered at a rate of 50 nL min^−1^ via a microsyringe pump attached to a glass pipette. The injection coordinates for the viruses (in millimeters, relative to the midline, bregma, and dorsal surface) were precisely followed: for the APC (±2.75, 1.70, −4.65) and for the LH (±0.95, −1.34, −5.25). Following the injection, the glass pipettes remained in position for a duration of 10 min to facilitate diffusion before being withdrawn. The mice were allowed to recover from anesthesia on a heated blanket and were then injected with antibiotics (ceftriaxone sodium, 0.1 g kg^−1^, i.p.) for 3 days to prevent infection. Each mouse was housed separately and given the opportunity to recuperate, allowing at least 3 weeks after surgery for the virus to be expressed.

To inhibit APC glutamatergic neurons, WT mice were bilaterally injected with AAVs containing the CaMK2α promoter encoding hM4Di (AAV‐CaMK2α‐hM4Di‐mCherry, 4 × 10^12^ vector genomes (vg) mL^−1^)) at a volume of 300 nL per side, or mCherry alone (AAV‐CaMK2α–mCherry, 4 × 10^12^ vg mL^−1^) in the same volume (as the control).

To inhibit APC^CRH^ neurons, Crh‐IRES‐Cre mice were bilaterally injected with a Cre‐dependent AAV vector containing hM4Di (AAV‐DIO‐hM4Di‐mCherry, 4 × 10^12^ vg mL^−1^) at a volume of 300 nL per side, or mCherry alone (AAV‐DIO‐mCherry, 4 × 10^12^ vg mL^−1^) in the same volume (as the control).

To activate APC^CRH^ neurons without side effect of epileptiform convulsions in mice due to over‐activation, Crh‐IRES‐Cre mice were unilaterally injected with a Cre‐dependent vector containing hM3Dq (AAV‐DIO‐hM3Dq‐mCherry, 5 × 10^12^ vg mL^−1^) at a volume of 300 nL, or mCherry alone (AAV‐DIO‐mCherry, 5 × 10^12^ vg mL^−1^) in the same volume (as the control).

For calcium signal recording, the APC of Crh‐IRES‐Cre mice was bilaterally injected with a Cre‐dependent AAV vector containing jGCaMP7s (AAV2/9‐hSyn‐FLEX‐GCaMP7s‐WPRE‐pA, 8 × 10^12^ vg mL^−1^) at a volume of 300 nL.

For GCN2 knock‐down in APC^CRH^ neurons and glutamatergic neurons, Crh‐IRES‐Cre and vGlut2‐IRES‐Cre mice were bilaterally injected with a Cre‐dependent AAV vector containing the mir‐30‐shGCN2 coding sequence and GFP protein in the opposite orientation, flanked by two inverted loxP sites (AAV9‐CMV‐bGiobin‐FLEX‐mir‐30‐shGCN2‐GFP; 5 × 10^12^ vg mL^−1^), at a volume of 300 nL into the APC, or an AAV vector containing the mir‐30‐scramble and GFP protein in the opposite orientation, flanked by two inverted loxP sites (AAV9‐CMV‐bGiobin‐FLEX‐mir‐30‐scramble‐GFP; dilute to 5 × 10^12^ vg mL^−1^), as a control. The target sequence 5ʹTCTGGATGGATTAGCTTATA‐3ʹ for GCN2 has been previously validated.^[^ ^36^ ^]^ Mice were given a 6‐week recovery period after surgery before experiments to ensure optimal efficiency of GCN2 knockdown.

For the GCN2 ablation study, GCN2 loxp/loxp (GCN2^+/+^) mice were bilaterally injected into the APC with an AAV vector containing a cassette expressing Cre recombinase protein with GFP (AAV9‐hSyn‐Cre‐GFP, 3.5 × 10^12^ vg mL^−1^) at a volume of 300 nL for each side, or an AAV vector containing a cassette expressing GFP protein (AAV9‐hSyn‐GFP; 3.5 × 10^12^ vg mL^−1^) in the same volume as the control. Mice were also given a 6‐week postoperative recovery period.

To inhibit the APC innervating the LH, WT mice were bilaterally injected with retroAAV under the hSyn promoter encoding Cre (retroAAV‐EGFP‐2A‐Cre, 1 × 10^13^ vg mL^−1^) into the LH at a volume of 300 nL per side, and AAV‐DIO‐hM4Di‐mCherry or AAV‐DIO‐mCherry virus (both 5 × 10^12^ vg mL^−1^, 300 nL) was bilaterally injected into the APC of the same mice.

For activation of the axon terminals of the APC^CRH^ neurons that project into the LH, Crh‐IRES‐Cre mice were unilaterally injected with a Cre‐dependent AAV vector containing hM3Dq (AAV‐EF1α‐DIO‐hM3Dq‐ mCherry ‐WPRE, 5 × 10^12^ vg mL^−1^) into the APC at a volume of 300 nL. 3 weeks later, guide cannulas (internal diameter, 1.25 mm) were implanted into the LH. The mice were allowed to recover for 1 week prior to the experiments.

### Histological Analysis

BAT was fixed in 4% paraformaldehyde (PFA) for 14–16 h. The tissues were then embedded in paraffin and the entire prepared block was cut into 8‐µm sections. After deparaffinization and rehydration, the sections were stained with H&E.

### Measurement of Metabolic Parameters

The body composition of mice was detected by a Bruker Minispec mq10 NMR Analyzer (Bruker, Billerica, MA, USA). BAT surface temperature was measured by an infrared camera (Magnity Electronics Co., Ltd., Shanghai, China). At least 3 days prior to the experiment, the mice were anesthetized, and the hair on their dorsal scapular area was carefully shaved off using a razor to fully expose the skin covering BAT. This allowed infrared camera to accurately capture the local surface temperature of the BAT. The rectal temperature of mice was measured using a digital thermometer (Physitemp Instruments, Clifton, NJ, USA). Indirect calorimetry was measured by metabolic cage (CLAMS‐16; Columbus Instruments, USA). NE and cAMP levels in the BAT were determined by ELISA kits (Nanjing Jiancheng Bioengineering Institute, Nanjing, China) in accordance with the use instructions.

### Immunofluorescence Staining Assays

Mice underwent transcardial perfusion with saline, and then perfused with phosphate‐buffered saline (PBS) containing 4% PFA. The mouse brains were dissected and fixed for an entire night at 4 °C in 4% PFA, and then underwent cryoprotection in PBS containing 20% and 30% sucrose at 4 °C. Free‐floating sections (25 µm) were generated using a cryostat. The slices were blocked for 1 h at room temperature in PBST (0.3% Triton X‐100) along with 5% normal donkey serum, followed by incubation with primary antibodies at 4 °C throughout the night and incubation with secondary antibodies at room temperature for 2 h. The primary antibodies utilized in the IF experiments comprised anti‐c‐Fos (1:1000, #2250), anti‐CaMK2α (1:1000, #50 049), and anti‐phosphate‐eIF2α (1:1000, #3398; all from Cell Signaling Technology); anti CRH (1:500, A1122) and anti GCN2 (1:1000, A12618; both from ABclonal); and anti GAD67 (1:500, MAB5406B, from Milipore).

### RNA Isolation and Relative Quantitative Real‐Time PCR

RNA was extracted by TRIzol reagent (Invitrogen, CA, USA). mRNA was reverse transcribed by using a High‐Capacity cDNA Reverse Transcription Kit (Thermo Scientific, CA, USA) and subjected to quantitative real‐time PCR using SYBR Green I Master Mix reagent on an ABI 7900 system (Applied Biosystems, CA, USA).

### Western Blot Analysis

Western blotting was performed as previously described.^[^ ^32^ ^]^ Tissue extracts were then immune‐blotted with the following primary antibodies: anti‐UCP1 (1:10000, Abcam, Cambridge, UK), anti‐TH (1:1000, Merck Millipore, Frankfurter, GER), anti‐β‐ACTIN (1:1000, Sigma, MO, USA), anti‐Phospho‐PKA Substrates (1:1000, Cell Signaling Technology) and anti‐TUBULIN (1:2500, Proteintech). Band intensities were measured using an e‐BLOT Touch Imager (Shanghai Yibote Optoelectronic Technology Co., Ltd., China).

### NE Turnover

In accordance with the prior study,^[^ ^33^ ^]^ α‐MPT was administered via i.p. injection at a dose of 100 mg kg^−1^, whereas the baseline group received an equivalent volume of saline. 2 h post‐administration, BAT was harvested for the detection of NE concentration. The turnover of NE was indicated by the reduction in NE levels observed in the α‐MPT‐treated group relative to the baseline group.

### APC Amino Acid Measurement

APC tissues were isolated and homogenized in 0.1N HCl on ice. Subsequently, the tissues were centrifuged at 5000 ×g for 5 min and the supernatant was evaporated to dryness. HCl (20 µL of 0.1 N) was added to the residue and then mixed for measurement. The amino acids in the APC were analyzed by Beijing MS Medical Research Co. Ltd (Beijing, China) using high‐performance liquid chromatography (Ultimate 3000, USA) and tandem mass spectrometry (API 3200 Q‐TRAP, USA).

### Chemogenetic Manipulation In Vivo

CNO (MedChemExpress, NJ, USA) dissolved in saline was injected (i.p.) into mice expressing hM4Di and hM3Dq in the target regions. The CNO dose was 3 mg kg^−1^ of body weight. Injections of CNO or saline were performed every 12 h for 3 d.

To activate the axon terminals of APC^CRH^ neurons projecting into the LH, a stainless‐steel injector, connected to a 10‐µL syringe and an infusion pump, was inserted into the guide cannula. CNO (1 µL, 5.8 µM) was infused at a rate of 500 nL min^−1^ under the control of a micro‐injection pump (RWD Life Science). The injector was withdrawn slowly 2 min after infusion, and thermo‐parameter measurements were conducted before and after micro‐injection.

### Whole‐Cell Patch Clamp Recording

Mice were anesthetized with isoflurane, and their brains were submerged in an oxygenated ice‐cold cutting solution containing (in mM) 26 NaHCO_3_, 2.5 KCl, 2 MgSO_4_, 2 CaCl_2_, 1.25 NaH_2_PO_4_∙2H_2_O, 10 D‐glucose, 213 sucrose at 315 mOsm, pH 7.4. The coronal APC brain sections (300 µm in thickness) were cut using a Leica VT1200s vibratome; collected in oxygenated artificial cerebrospinal fluid (ACSF) solution containing (in mM) 126 NaCl, 3.5 KCl, 1.25 NaH_2_PO_4_∙2H_2_O, 26 NaHCO_3_, 2 MgSO_4_, 2 CaCl_2_, and 25 D‐glucose at 33 °C; and incubated for 30 min. The sections were subsequently transferred to room temperature for additional utilization. During the recording process, an APC section was positioned into the recording chamber, which was perfused with oxygenated ACSF, and whole‐cell recordings were executed. The acquired signals were amplified using a MultiClamp 700 B amplifier (Molecular Devices) and then digitized at a rate of 20 kHz through a DigiData 1550 B (Molecular Devices). Stimulation and recording were facilitated by Clampex 11.0 software (Axon Instruments). The intracellular solution employed for current‐clamp recording comprised (in mM) 140 K‐gluconate, 3 KCl, 2 MgCl_2_ ∙6H_2_O, 10 HEPES, 2 Na_2_ATP, and 0.2 EGTA at 315 mOsm and a pH of 7.4. The recorded data were analyzed utilizing Clampfit software (Molecular Devices).

Current‐clamp recordings were conducted to determine the intrinsic excitability of APC^CRH^ cells. We recorded the voltage changes of the cells in response to a series of 500 ms current steps ranging from −60 to 300 pA, with increments of 20 pA. As described previously,^[^ ^41^ ^]^ the intrinsic properties of each cell, such as the rheobase and resting potential, were characterized. The rheobase activity was described as the minimum current that triggered the first action potential. The resting potential was measured immediately after rupturing the cell membrane, with a holding current of 0 pA. The input resistance was obtained from the membrane test window of the software following membrane break‐in during recording (Clampex 11.0).

### Calcium Imaging

Mice were anesthetized with isoflurane and their brains were submerged in oxygenated ice‐cold cutting solution. Then, coronal APC brain sections (300 µm in thickness) were cut and collected in oxygenated ACSF solution, as described above. The brain slices were treated as follows: 3‐min incubation with ACSF containing 100 µM leucine served as the baseline, followed by an 8‐min washout period using ACSF devoid of leucine. Images were acquired using an upright microscope (FVMPE‐RS, Evident powered by OLYMPUS) equipped with a femtosecond laser (Ultrafast Oscillators, InSight X3, Spectra‐Physics) and a 25× objective via photomultiplier tubes (FV30‐RXD) using FV31S‐SW Viewer software. For GCaMP7s imaging, 920‐nm excitation and a green barrier filter (495 nm−540 nm) were used. Images were acquired at a frame rate of 0.919 Hz and 512 × 512 pixels. All images were analyzed using ImageJ (NIH) and MATLAB (MathWorks). The dF/F ratio was calculated as: (F‐F0)/F0, where F0 was the average 488 Changes fluorescence intensity of baseline. Neurons with mean fluorescence signal increase exceeding the threshold of 10% dF/F were classified as leucine deprivation‐activated neurons. Conversely, neurons exhibiting a mean fluorescence signal decrease surpassing the threshold were categorized as leucine deprivation‐inhibited neurons, whereas neurons that did not meet either criterion were designated as leucine‐insensitive neurons.

### Statistical Analysis

Statistical analyses were conducted utilizing GraphPad Prism, version 8.0 (GraphPad Software, San Diego, CA, USA). All numerical values are reported as the mean, accompanied by the standard error of the mean (SEM). Comparisons between two groups were carried out using a two‐tailed unpaired Student's t‐test. For experiments necessitating multiple comparisons, the data underwent a two‐way analysis of variance (ANOVA), subsequently followed by Tukey's multiple comparisons test. Each histogram displays individual data points to illustrate the variability in the measurements. Significant differences emerging from the above tests are indicated in the Figures by *p < 0.05, **p < 0.01, ***p < 0.001, and ****p < 0.0001.

## Conflict of Interest

The authors declare no conflict of interest.

## Author Contributions

P.L. designed and carried out overall experiments, analyzed data, wrote, reviewed, and edited the manuscript. K.T., Y.G., and M.T. assisted with patch clamp experiments. K.T., Y.G., M.T., Y.N., K.L., S.N., S.W., and S.C. assisted with mouse experiments. X.J., H.J., F.X., W.L., and X.L. contributed to the discussion. F.Y. and F.G. directed the research, contributed to discussion, wrote, reviewed, and edited the manuscript.

## Supporting information



Supporting Information

## Data Availability

The data that support the findings of this study are available from the corresponding author upon reasonable request.
