# Supplementary material for: Amino Acid‐Sensing Neurons in the Anterior Piriform Cortex Control Brown Adipose Tissue Thermogenesis
Source: Adv Sci (Weinh). 2025 Apr 30;12(27):2502421. doi: 10.1002/advs.202502421 (PMC12279160; doi:10.1002/advs.202502421)
Supplement: Supplementary file 1 — Supporting Information [file ADVS-12-2502421-s001.docx]

Supporting Information

**Amino acid-sensing Neurons in the Anterior Piriform Cortex Control Brown Adipose Tissue Thermogenesis**

*Peixiang Luo^3^, Kexin Tong^2^, Yeting Gan^2^, Min Tang^2^, Yuguo Niu^2^, Kan Liu^3^, Shihong Ni^2^, Shangming Wu^3^, Xiaoxue Jiang^2^, Haizhou Jiang^1,2^, Fei Xiao^1,2^, Shanghai Chen^2^, Wei Lv^3^, Xiaoying Li^1^, Feixiang Yuan^1,2^* and Feifan Guo^1,2^**

**
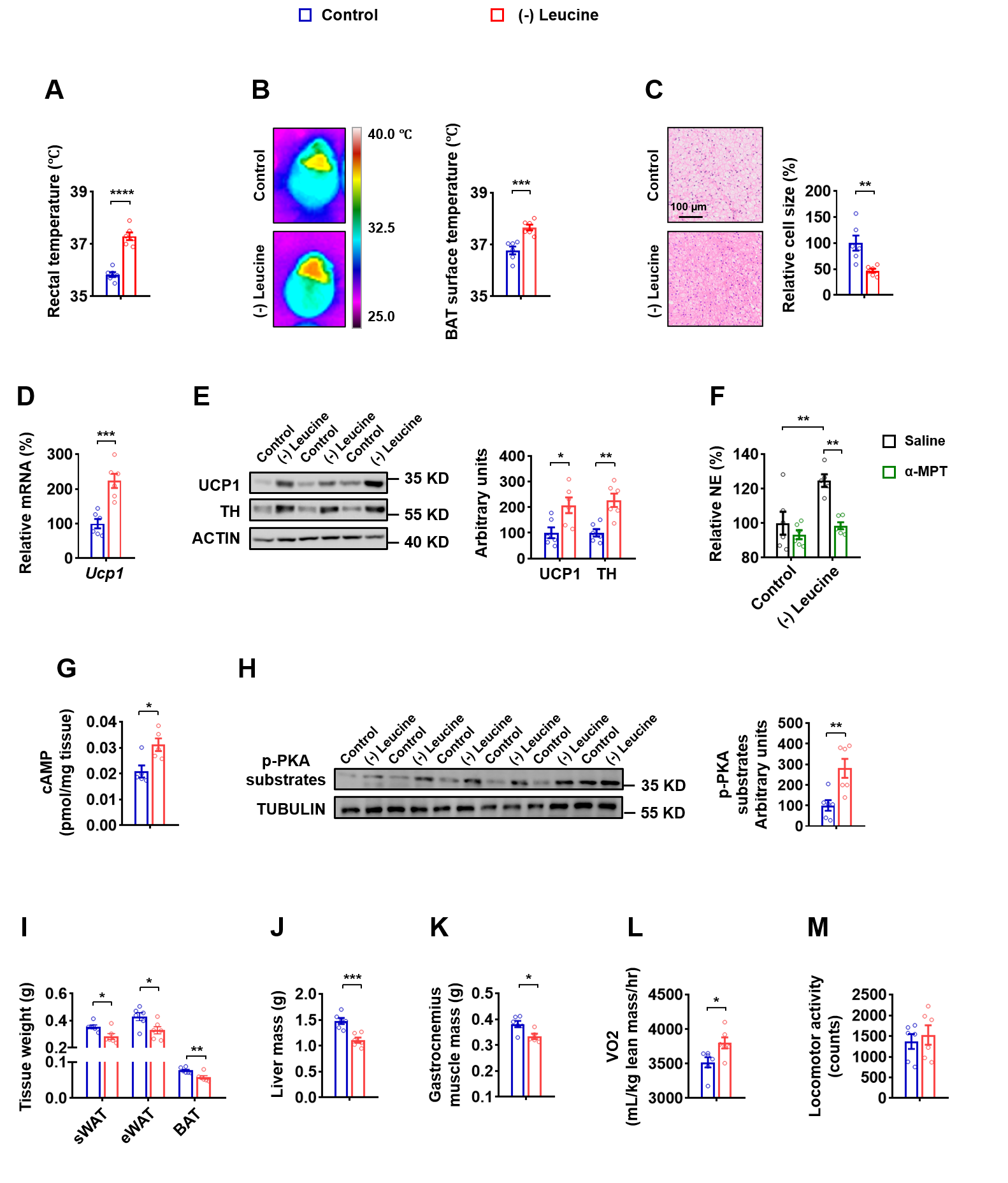
**

**Figure S1. Leucine deprivation enhances BAT thermogenesis.** 8- to 12-week-old male wild-type (WT) mice fed a control (Control) or leucine-deficient [(–) Leucine] diet for 3 days. A) Rectal temperature (n = 6 per group). B) Representative infrared thermal images (left) and quantifications (right, n = 6 per group). C) Representative images of hematoxylin and eosin (H&E) staining of BAT (left) and BAT cell size quantified by Image J analysis of H&E images (right, n = 6 per group). D) *Ucp1* mRNA in BAT (n = 6 per group). E) UCP1 and TH protein in BAT (left) and quantifications (right, n = 6 per group). F) NE content in BAT with i.p. injection of saline or α-MPT (n = 5–6 per group). G) cAMP level in BAT (n = 5 per group). H) p-PKA substrates protein in BAT (left) and quantifications (right, n = 6 per group). I) Tissue weight of subcutaneous white adipose tissue (sWAT), epididymal white adipose tissue (eWAT) and BAT (n = 6 per group). J) Tissue weight of liver (n = 6 per group). K) Tissue weight of gastrocnemius muscle (n = 5–6 per group). L) 24-h oxygen consumption normalized by lean mass measured by the comprehensive lab animal monitoring system (n = 6 per group). M) Locomotor activity (n = 6 per group). Data are represented as mean ± SEM. Statistical analyses were performed by two-tailed unpaired Student’s t test; **p* < 0.05, ***p* < 0.01 and ****p* < 0.001.


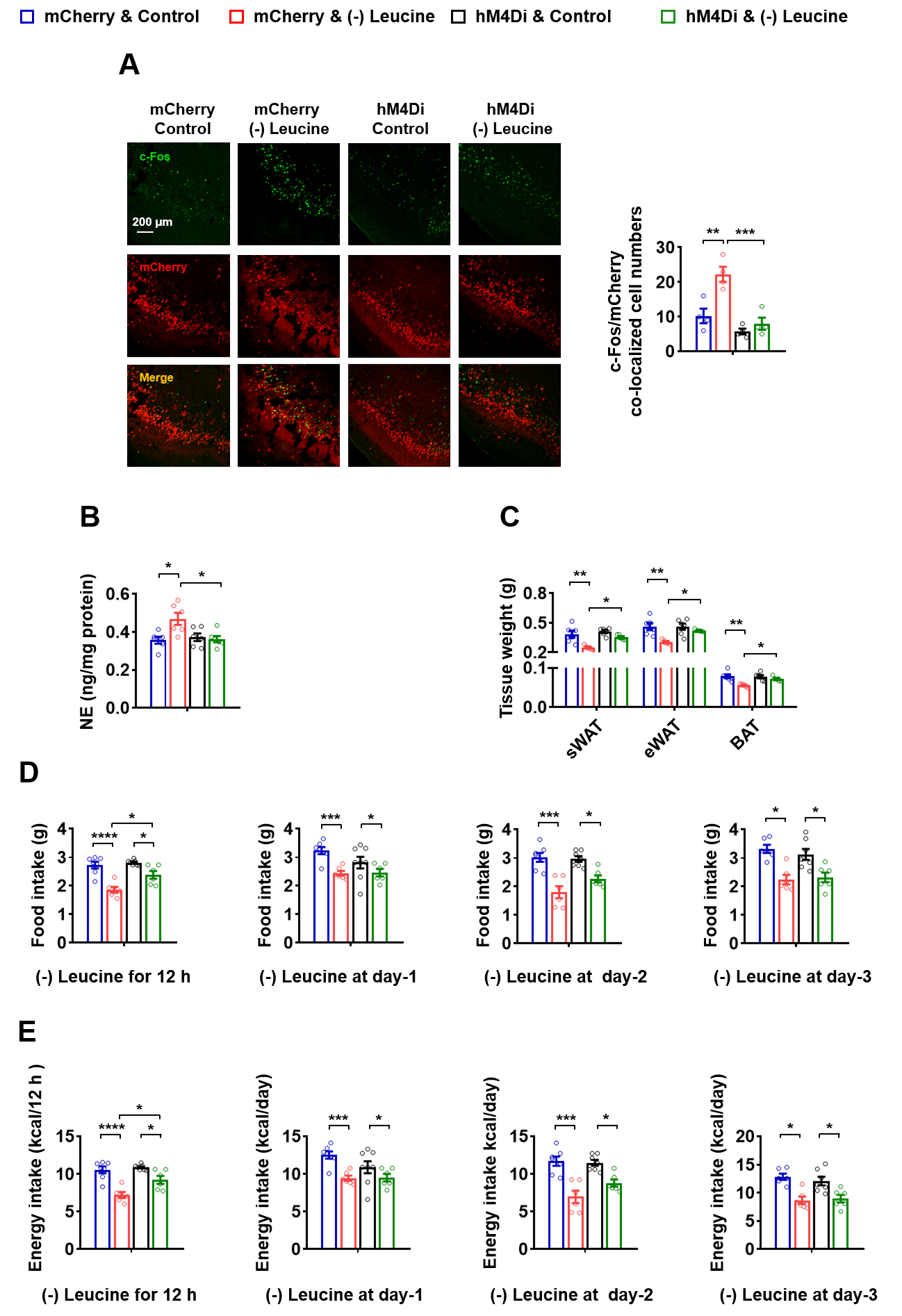


**Figure S2. Metabolic parameters related to mice with inhibition of APC glutamatergic neuronal activity.** 8- to 12-week-old male WT mice receiving AAVs expressing CaMK2α-mCherry (mCherry) or CaMK2α-hM4Di (hM4Di), all received CNO injections every 12 h for 3 days, simultaneously fed a Control or (–) Leucine diet for 3 days. A) Immunofluorescence (IF) staining for c-Fos (green), mCherry (red) and merge (yellow) in APC sections (left), and quantifications of c-Fos and mCherry colocalized cell numbers (right, n = 4 per group). B) NE levels in BAT (n = 6 per group). C) Tissue weight of sWAT, eWAT and BAT (n = 5–6 per group). D) Food intake (n = 6–7 per group). E) Energy intake (n = 6–7 per group). Data are represented as mean ± SEM. Statistical analyses were performed by two-way ANOVA with Tukey’s multiple comparisons test; **p* < 0.05, ***p* < 0.01, ****p* < 0.001 and **** *p* < 0.0001.


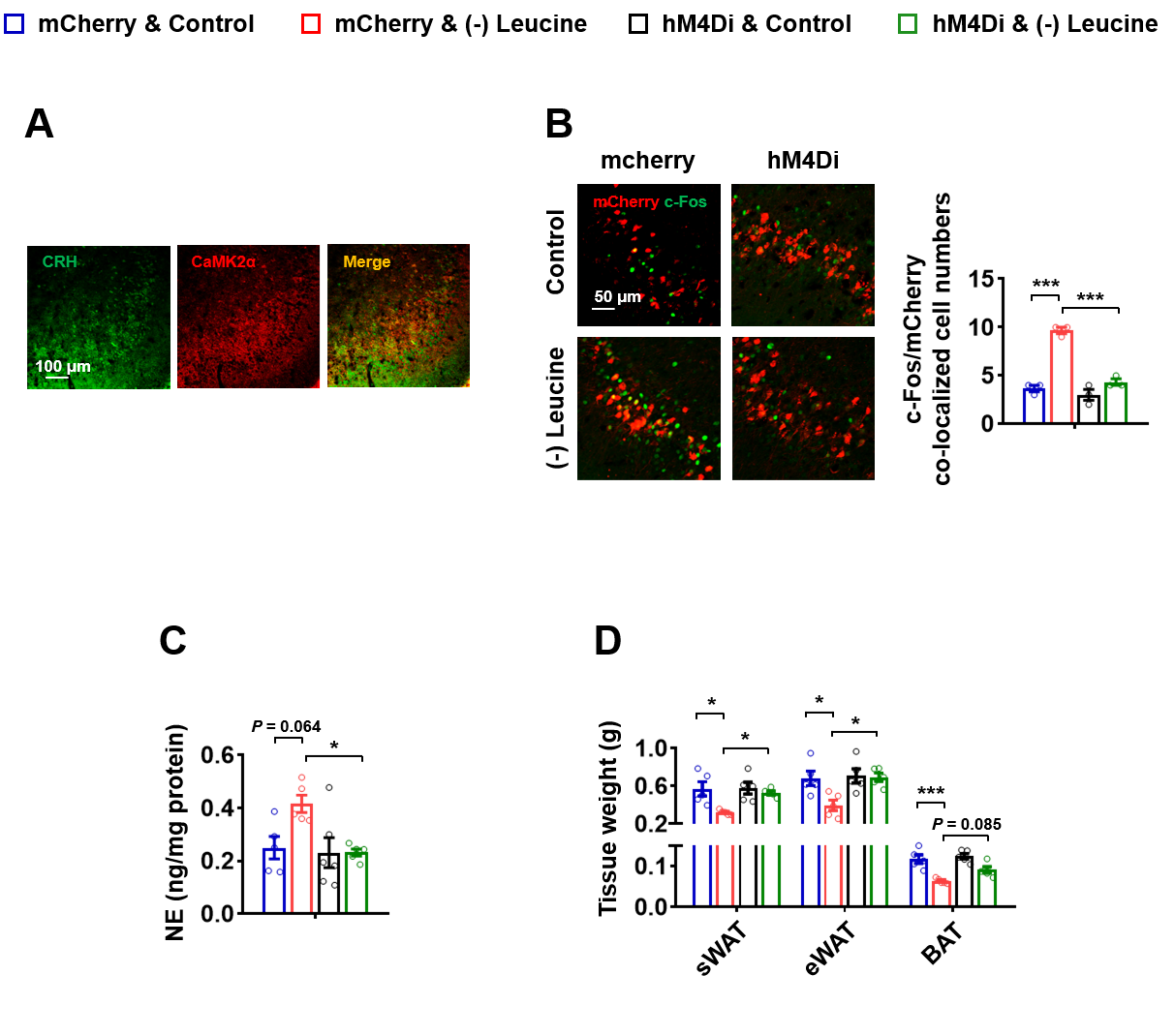


**Figure S3. Metabolic parameters related to mice with inhibition of APC CRH neuronal activity.** 8- to 12-week-old male Crh-IRES-Cre mice receiving AAVs expressing DIO-mCherry (mCherry) or DIO-hM4Di (hM4Di), all received CNO injections every 12 h for 3 days, simultaneously fed a Control or (–) Leucine diet for 3 days. A) IF staining for CaMK2α (red), CRH (green) and merge (yellow) in APC sections. B) IF staining for mCherry (red), c-Fos (green) and merge (yellow) in APC sections (left), and quantifications of c-Fos and mCherry colocalized cell numbers (right, n = 3 per group). C) NE levels in BAT (n = 5 per group). D) Tissue weight of sWAT, eWAT and BAT (n = 5 per group). Data are represented as mean ± SEM. Statistical analyses were performed by two-way ANOVA with Tukey’s multiple comparisons test; **p* < 0.05, ***p* < 0.01 and ****p* < 0.001.


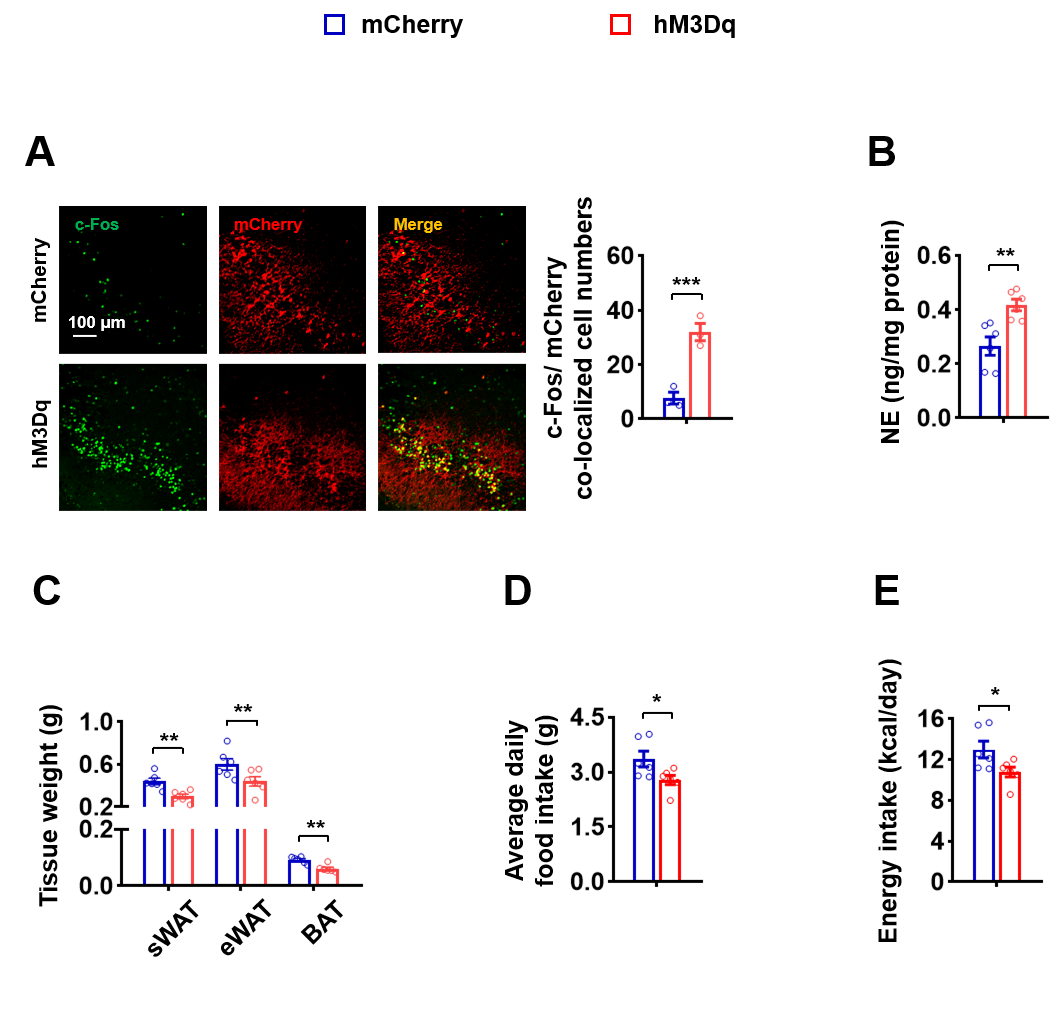


**Figure S4. Metabolic parameters related to mice with activation of APC CRH neuronal activity.** 8- to 12-week-old male Crh-IRES-Cre mice receiving AAVs expressing DIO-mCherry (mCherry) or DIO-hM3Dq (hM3Dq), all received CNO injections every 12h for 3 days, simultaneously fed a normal chow diet. A) IF staining for c-Fos (green), mCherry (red) and merge (yellow) in APC sections (left), and quantifications of c-Fos and mCherry colocalized cell numbers (right, n = 3 per group). B) NE levels in BAT (n = 6 per group). C) Tissue weight of sWAT, eWAT and BAT (n = 6 per group). D) Daily food intake (n = 6 per group). E) Daily energy intake (n = 6 per group). Data are represented as mean ± SEM. Statistical analyses were performed by two-tailed unpaired Student’s t test; **p* < 0.05, ***p* < 0.01 and ****p* < 0.001.

**
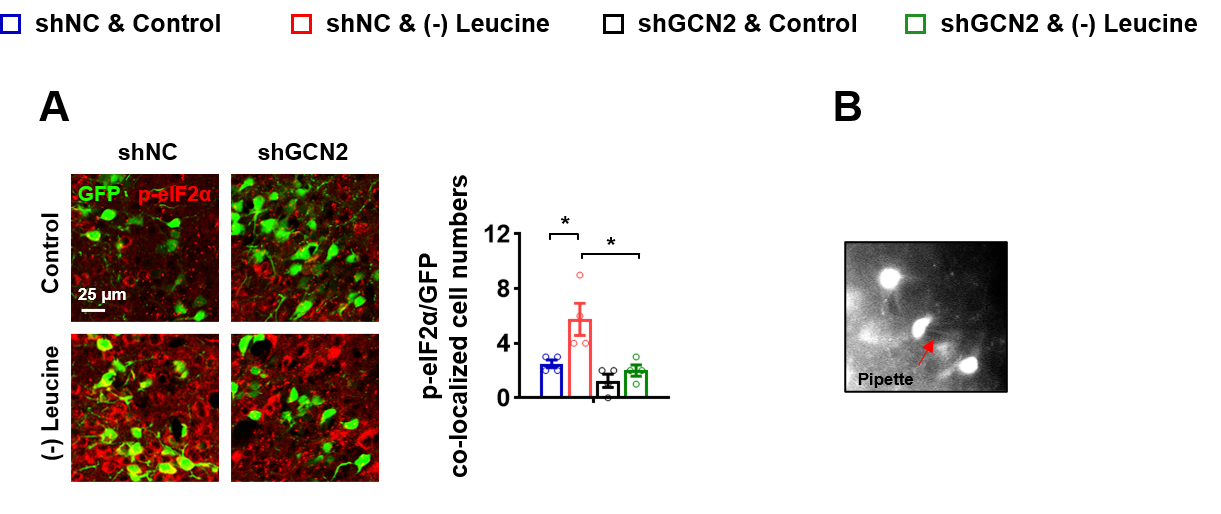
**

**Figure S5. The efficiency of GCN2 knockdown in APC^CRH^ neurons.** 8- to 12-week-old male Crh-IRES-Cre mice receiving AAVs expressing FLEX-shNC-GFP (shNC) or FLEX-shGCN2 GFP (shGCN2), all fed a Control or (–) Leucine diet for 3 days. A) IF staining for phosphorylation at serine 51 site of eukaryotic translation initiation factor 2 subunit 1 (p-eIF2α) (red), GFP (green) and merge (yellow) in APC sections (left), and quantifications (right, n = 3 per group). B) A differential interference contrast (DIC) image showing a recorded CRH-GFP cell in APC brain sections. Data are represented as mean ± SEM. Statistical analyses were performed by two-way ANOVA with Tukey’s multiple comparisons test; **p* < 0.05.


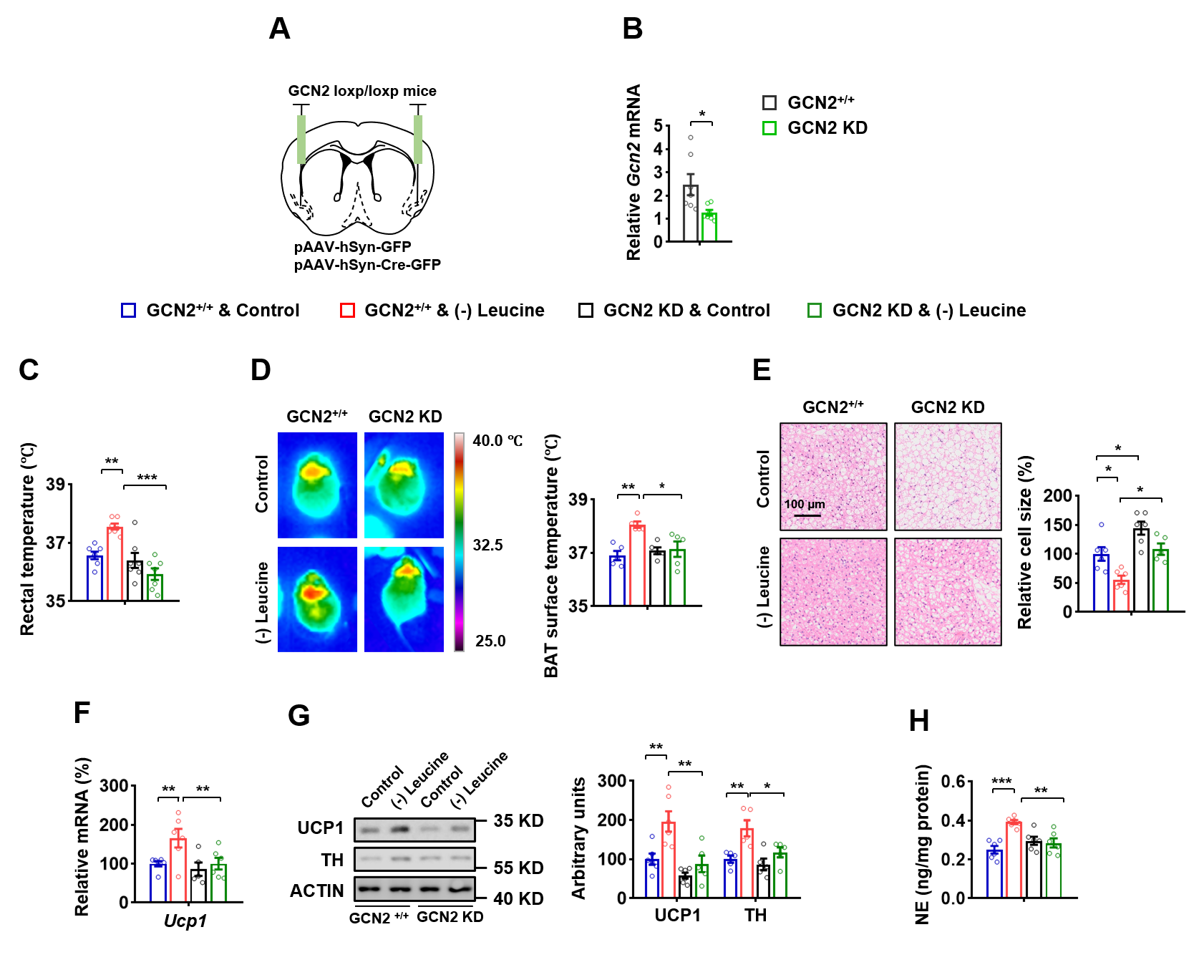


**Figure S6. GCN2 knockdown in APC is necessary for BAT thermogenesis induced by leucine deprivation.** 8- to 12-week-old male GCN2 loxp /loxp mice receiving AAVs expressing Cre-GFP (GCN2 KD) or GFP (GCN2^+/+^) into the APC, all fed a Control or (–) Leucine diet for 3 days. A) Schematics of virus-mediated Cre expression (green) sites. B) *Gcn2* mRNA in APC (n = 7 per group). C) Rectal temperature (n = 7 per group). D) Representative infrared thermal images (left) and the quantifications (right, n = 5 per group). E) Representative images of H&E staining of BAT (left) and quantifications (right, n = 6 per group). F) *Ucp1* mRNA in BAT (n = 5–6 per group). G) UCP1 and TH protein levels in BAT (left) and quantifications (right, n = 5 per group). H) NE levels in BAT (n = 6 per group). Data are represented as mean ± SEM. Statistical analyses were performed by two-tailed unpaired Student’s t test for (B) or by two-way ANOVA with Tukey’s multiple comparisons test for (C–H); **p* < 0.05, ***p* < 0.01 and ****p* < 0.001.


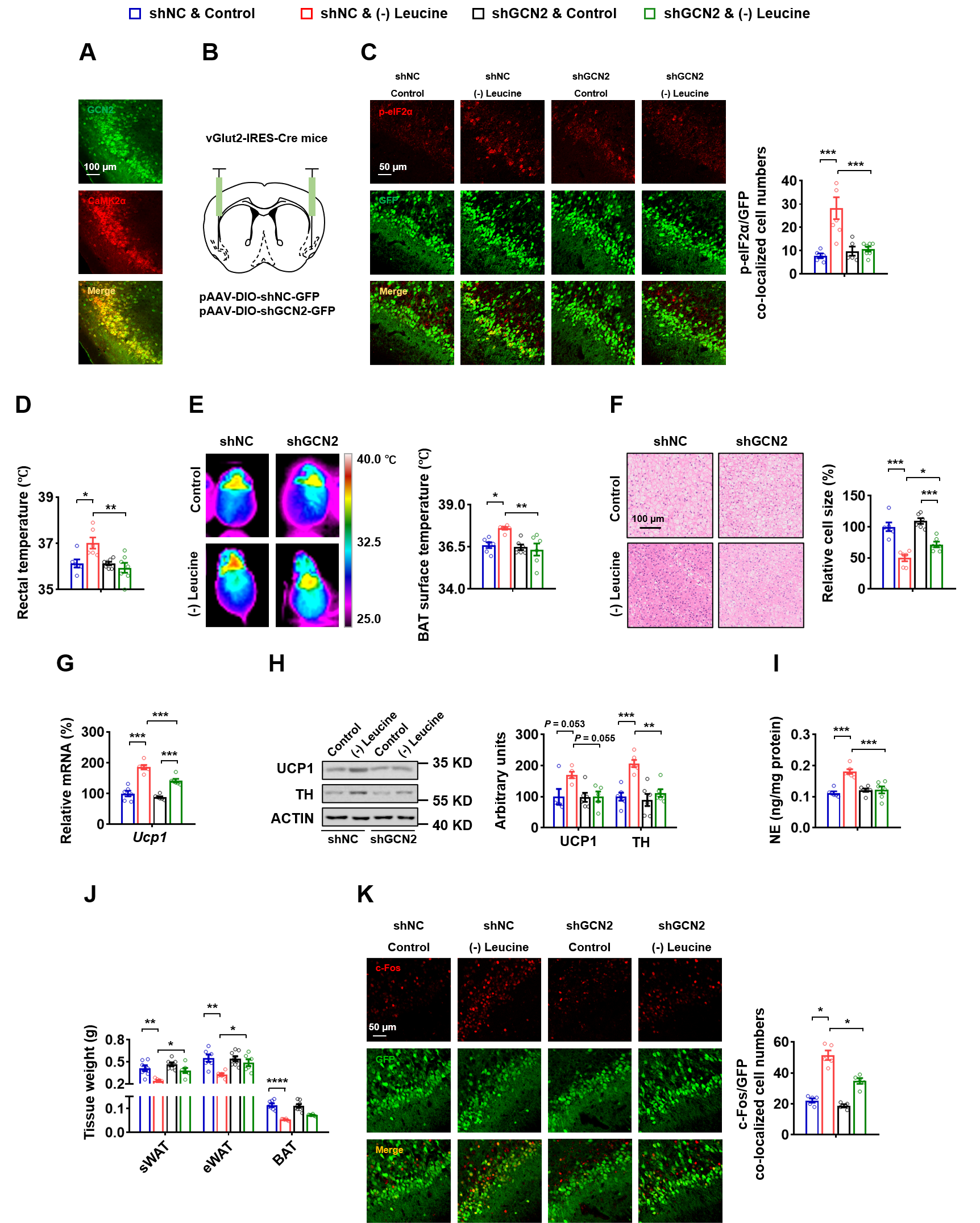


**Figure S7. GCN2 knockdown in APC glutamatergic neurons is necessary for BAT thermogenesis induced by leucine deprivation.** 8- to 12-week-old male vGlut2-IRES-Cre mice receiving AAVs expressing FLEX-shNC-GFP (shNC) or FLEX-shGCN2 GFP (shGCN2), all fed a Control or (–) Leucine diet for 3 days. A) IF staining for CaMK2α (red), GCN2 (green) and merge (yellow) in APC sections. B) Schematics of virus-mediated shGCN2 expression (green) sites. C) IF staining for p-eIF2α (red), GFP (green) and merge (yellow) in APC (left), and quantifications of p-eIF2α and GFP colocalized cell numbers (right, n = 5–7 per group). D) Rectal temperature (n = 6–7 per group). E) Representative infrared thermal images (left) and the quantifications (right, n = 6 per group). F) Representative images of H&E staining of BAT (left) and BAT cell size quantified by Image J analysis of H&E images (right, n = 6 per group). G) *Ucp1* mRNA in BAT (n= 6 per group). H) UCP1 and TH protein levels in BAT (left) and quantified by densitometric analysis (right, n = 5–6 per group). I) NE levels in BAT (n = 6 per group). J) Tissue weight of sWAT, eWAT and BAT (n = 6–8 per group). K) IF staining for c-Fos (red), GFP (green) and merge (yellow) in APC (left), and quantifications of c-Fos and GFP colocalized cell numbers (right, n = 4–5 per group). Data are represented as mean ± SEM. Statistical analyses were performed by two-way ANOVA with Tukey’s multiple comparisons test; **p* < 0.05, ***p* < 0.01, ****p* < 0.001 and **** *p* < 0.0001.


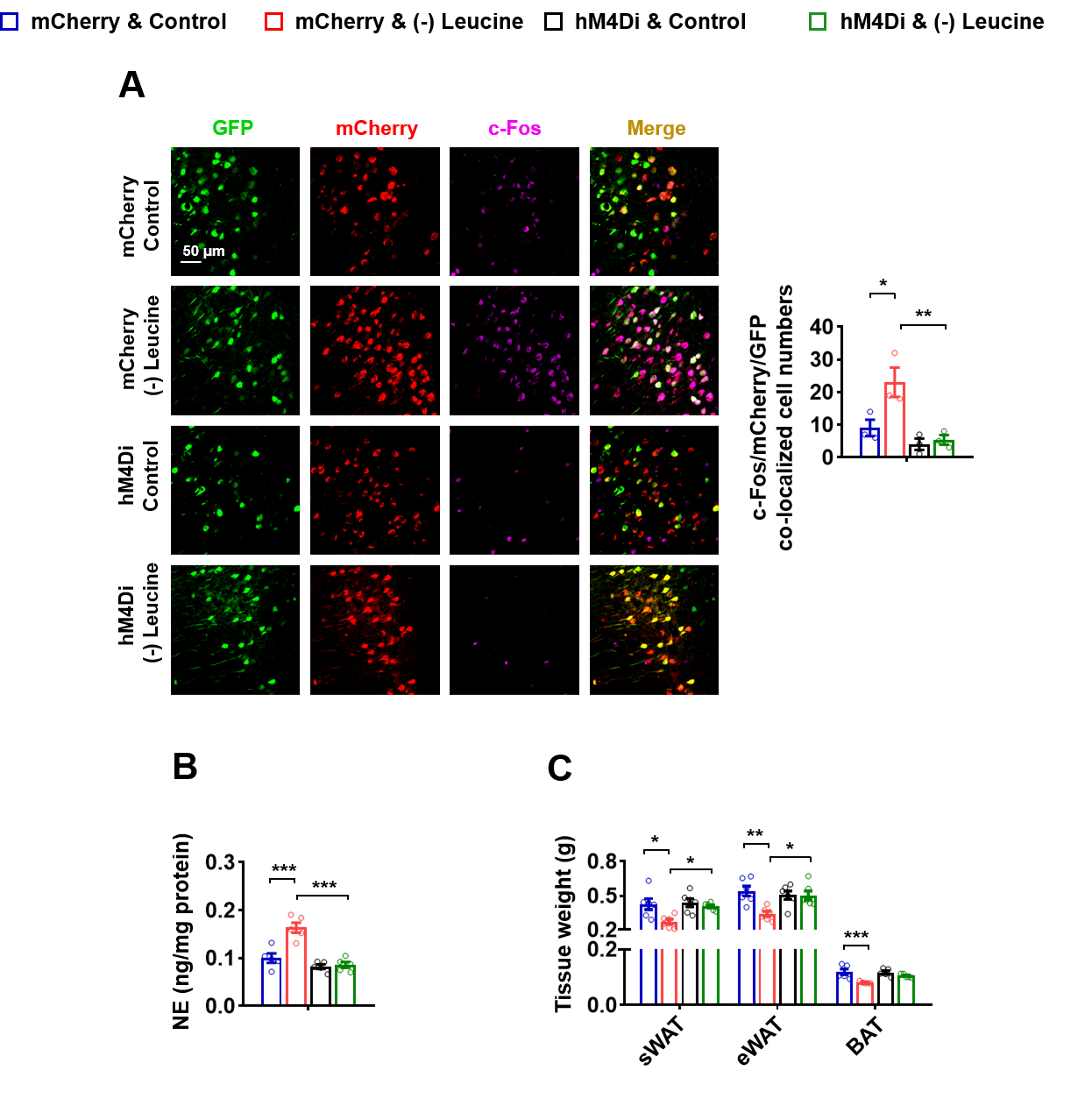


**Figure S8.** **Metabolic parameters related to mice with inhibition of the APC-LH circuit.** 8- to 12-week-old male WT mice receiving retroAAV-EGFP-2A-Cre AAV to the LH and Cre-dependent AAV-DIO-hM4Di-mCherry or control AAV to the APC. All received CNO injections every 12 h for 3 days, simultaneously fed a Control or (–) Leucine diet for 3 days. A) IF staining for GFP (green), mCherry (red), c-Fos (cyan) and merge (white) in APC sections (left), and quantifications of c-Fos, mCherry and GFP colocalized cell numbers (right, n = 3 per group). B) NE levels in BAT (n = 5 per group). C) Tissue weight of sWAT, eWAT and BAT (n = 5–6 per group). Data are represented as mean ± SEM. Statistical analyses were performed by two-way ANOVA with Tukey’s multiple comparisons test; **p* < 0.05, ***p* < 0.01 and ****p* < 0.001.


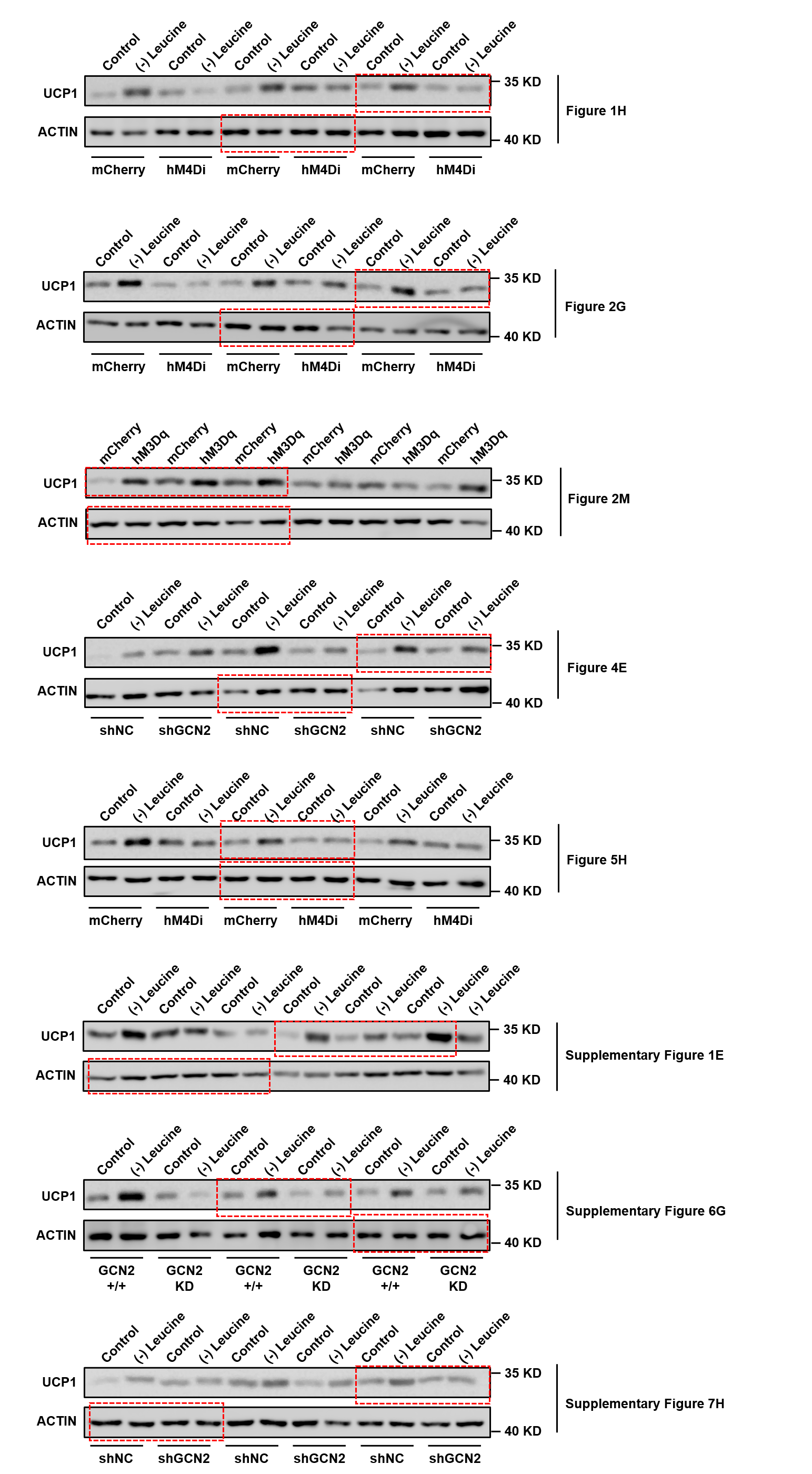


**Figure S9. Western blots full gel of UCP1 and ACTIN in related figures.** All blots in Figure 1H, 2G, 2M, 4E, 5H; Figure S1E, 6G, 7H respectively.

|  | Control Diet | | (–) Leucine Diet | |
| --- | --- | --- | --- | --- |
|  | gm (%) | kcal % | gm (%) | kcal % |
| Protein | 17.0 | 17.6 | 15.8 | 16.3 |
| Carbohydrate | 68.5 | 70.8 | 69.7 | 72.1 |
| Fat | 5.0 | 11.6 | 5.0 | 11.6 |
| kcal/gm |  | 3.87 |  | 3.87 |
| Ingredient (gm) |  |  |  |  |
| L-Arginine | 10 | 40 | 10 | 40 |
| L-Histidine-HCl-H_2_O | 6 | 24 | 6 | 24 |
| L-Isoleucine | 8 | 32 | 8 | 32 |
| L-Leucine | 12 | 48 | 0 | 0 |
| L-Lysine-HCl | 14 | 56 | 14 | 56 |
| L-Methionine | 6 | 24 | 6 | 24 |
| L-Phenylalanine | 8 | 32 | 8 | 32 |
| L-Threonine | 8 | 32 | 8 | 32 |
| L-Tryptophan | 2 | 8 | 2 | 8 |
| L-Valine | 8 | 32 | 8 | 32 |
| L-Alanine | 10 | 40 | 10 | 40 |
| L-Asparagine-H_2_O | 5 | 20 | 5 | 20 |
| L-Aspartate | 10 | 40 | 10 | 40 |
| L-Cystine | 4 | 16 | 4 | 16 |
| L-Glutamic Acid | 30 | 120 | 30 | 120 |
| L-Glutamine | 5 | 20 | 5 | 20 |
| Glycine | 10 | 40 | 10 | 40 |
| L-Proline | 5 | 20 | 5 | 20 |
| L-Serine | 5 | 20 | 5 | 20 |
| L-Tyrosine | 4 | 16 | 4 | 16 |
| Total L-Amino Acids | 170 | 0 | 158 | 0 |
| Corn Starch | 550.5 | 2202 | 562.5 | 2250 |
| Maltodextrin 10 | 125 | 500 | 125 | 500 |
| Cellulose | 50 | 0 | 50 | 0 |
| Corn Oil | 50 | 450 | 50 | 450 |
| Mineral Mix S10001 | 35 | 0 | 35 | 0 |
| Sodium Bicarbonate | 7.5 | 0 | 7.5 | 0 |
| Vitamin Mix V10001 | 10 | 40 | 10 | 40 |
| Choline Bitrartrate | 2 | 0 | 2 | 0 |
| Red Dye, FD&C #40 | 0 | 0 | 0.025 | 0 |
| Blue Dye, FD&C #1 | 0.05 | 0 | 0 | 0 |
| Yellow Dye, FD&C #5 | 0 | 0 | 0.025 | 0 |
|  |  |  |  |  |
| Total | 1000.05 | 3872 | 1000.05 | 3872 |

**Table S1. Composition of control diet and leucine-deficient diet.**
